# Dissection of GTPase-activating proteins reveals functional asymmetry in the COPI coat of budding yeast

**DOI:** 10.1242/jcs.232124

**Published:** 2019-08-29

**Authors:** Eric C. Arakel, Martina Huranova, Alejandro F. Estrada, E-Ming Rau, Anne Spang, Blanche Schwappach

**Affiliations:** 1 Department of Molecular Biology, Universitätsmedizin Göttingen, Humboldtallee 23, 37073 Göttingen, Germany; 2 Growth and Development, Biozentrum, University of Basel, Klingelbergstrasse 50/70, 4056 Basel, Switzerland; 3 Laboratory of Adaptive Immunity, Institute of Molecular Genetics of the Czech Academy of Sciences, Videnska 1083, 142 20 Prague 4, Czech Republic; 4 Max-Planck Institute for Biophysical Chemistry, 37077 Göttingen, Germany

**Keywords:** AMP kinase, Arf GTPase, ArfGAP, COPI vesicle, Gcs1, Glo3

## Abstract

The Arf GTPase controls formation of the COPI vesicle coat. Recent structural models of COPI revealed the positioning of two Arf1 molecules in contrasting molecular environments. Each of these pockets for Arf1 is expected to also accommodate an Arf GTPase-activating protein (ArfGAP). Structural evidence and protein interactions observed between isolated domains indirectly suggest that each niche preferentially recruits one of the two ArfGAPs known to affect COPI, i.e. Gcs1/ArfGAP1 and Glo3/ArfGAP2/3, although only partial structures are available. The functional role of the unique non-catalytic domain of either ArfGAP has not been integrated into the current COPI structural model. Here, we delineate key differences in the consequences of triggering GTP hydrolysis through the activity of one versus the other ArfGAP. We demonstrate that Glo3/ArfGAP2/3 specifically triggers Arf1 GTP hydrolysis impinging on the stability of the COPI coat. We show that the Snf1 kinase complex, the yeast homologue of AMP-activated protein kinase (AMPK), phosphorylates the region of Glo3 that is crucial for this effect and, thereby, regulates its function in the COPI-vesicle cycle. Our results revise the model of ArfGAP function in the molecular context of COPI.

This article has an associated First Person interview with the first author of the paper.

## Abstract (precis)

**Highlighted Article:** The regulatory domain of the COPI-associated ArfGAP Glo3 can stabilize the COPI coat. GTP hydrolysis is necessary to resolve the stabilised state. This mechanism is regulated by phosphorylation.

## INTRODUCTION

Coated vesicles transport proteins and lipids between compartments of the secretory pathway. Coats are macromolecular assemblies that associate with membranes, selectively capture proteins and lipids, deform the underlying membrane to form vesicles, and help accurately target these vesicles to their physiological destinations.

The COPI coat is formed by an obligate heptamer – also termed coatomer – consisting of α, β′, ε, β, γ, δ and ζ subunits, and is recruited *en bloc* to membranes (Hara-Kuge et al., 1994). Fundamentally, the COPI coat mediates the retrograde trafficking of proteins and lipids from the Golgi to the ER, and within intra-Golgi compartments (Arakel et al., 2016; Beck et al., 2009; Pellett et al., 2013; Spang and Schekman, 1998). Several reports have also implicated COPI in endosomal recycling and regulation of lipid droplet homeostasis (Aniento et al., 1996; Beller et al., 2008; Xu et al., 2017).

**Fig. 1.**
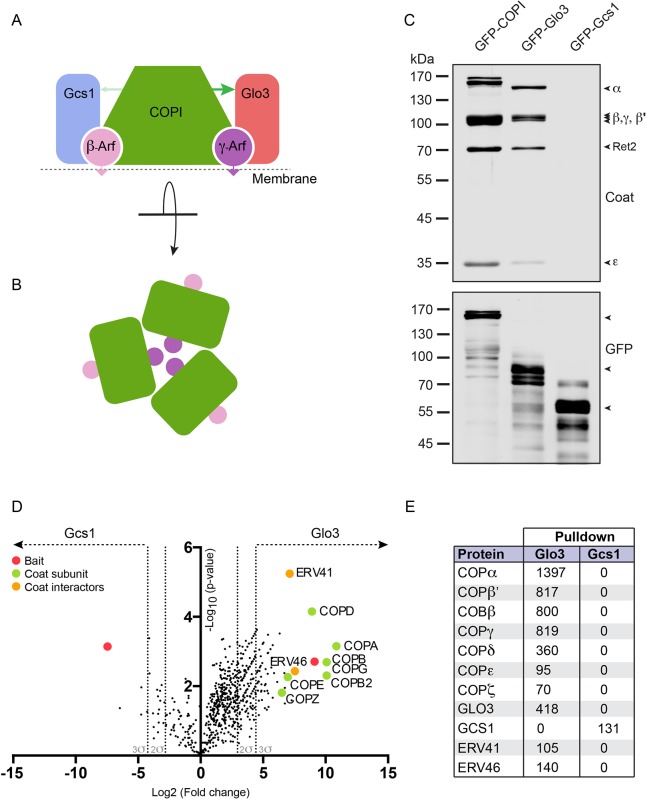
**COPI and Glo3 are stably associated.** (A) Schematic illustration of the heptameric COPI coat in complex with two Arf1 molecules (β-Arf and γ-Arf) and the two *S. cerevisiae* ArfGAPs (Glo3 and Gcs1). The thickness of the arrow indicates the differential affinity between COPI and the two ArfGAPs based on reports utilising isolated domains (Suckling et al., 2014; Watson et al., 2004). (B) Schematic illustration of the COPI triad, the symmetric basic unit of the coat. γ-Arf1 occupies the centre of a triad, whereas β-Arf1 lies at the periphery where the membrane surface is more exposed. (C) Affinity chromatography of GFP-tagged proteins isolated from the cytosol of the three indicated strains. Eluates were analysed by SDS-PAGE and western blotting. The blots were probed for coat subunits (top) or the respective GFP fusion protein (bottom). (D) Volcano plot analysis of proteins identified by mass spectrometry following the affinity chromatography of Glo3 and Gcs1 from detergent extracts of the indicated strains. The -log10 of the *P*-value indicating significance is plotted against the log2 of the enrichment. Coatomer subunits and the identified interaction partners that were significantly enriched (>3σ), are shown in green and orange, respectively. (E) List and spectral counts of interaction partners identified by mass spectrometry following the affinity chromatography of Glo3 and Gcs1 from detergent extracts of the indicated strains. COPI and Glo3 appear to be in a stable complex. Compare with Table S3 for a full list of co-purifying proteins.

Activation of the small GTPase Arf1 and its subsequent membrane anchoring by exchanging GDP with GTP through a guanine nucleotide exchange factor (GEF), promotes recruitment of coatomer to membranes (Antonny et al., 1997; Yu et al., 2012). The mammalian COPI-associated Arf1 GTPase-activating proteins (GAPs) ArfGAP1 and ArfGAP2/3, and their respective *Saccharomyces cerevisiae* homologues Gcs1 and Glo3, stimulate GTP hydrolysis in Arf1 (Spang et al., 2010; Weimer et al., 2008) (Fig. 1A,B). Inhibition of GTP hydrolysis results in deficient sorting and accumulation of COPI on the membrane (Lanoix et al., 1999; Nickel et al., 1998; Presley et al., 2002; Tanigawa et al., 1993). Hence, GTP hydrolysis in Arf1 is thought to be bi-functional, effecting efficient cargo capturing and vesicle uncoating.

Both ArfGAPs contain an evolutionarily conserved catalytic zinc finger domain, whereas their non-catalytical domains are structurally unrelated (Randazzo and Hirsch, 2004; Schindler et al., 2009; Spang et al., 2010). Why COPI relies on more than one ArfGAP is a mystery. There is circumstantial evidence suggesting that Gcs1/ArfGAP1 and Glo3/ArfGAP2/3 fulfil different functions in the COPI vesicle cycle – yet the precise role of either ArfGAP remains elusive (Poon et al., 1999, 2001; Schindler and Spang, 2007; Schindler et al., 2009). Curiously, as opposed to many other GAPs and effectors of GTPases, COPI and ArfGAP do not compete for access to Arf1. Instead, all three exist in one complex, and the activity of either ArfGAP is significantly enhanced in the presence of COPI (Szafer et al., 2001; Weimer et al., 2008). A series of primarily *in vitro* reconstitution assays have helped to elucidate the intricacies of this process, unequivocally demonstrating that both ArfGAP1 and ArfGAP2 can initiate COPI vesicle uncoating (Weimer et al., 2008).

However, little is known about the precise orchestration of GTP-hydrolysis in Arf1, which governs COPI function. The specific roles of the two COPI-associated ArfGAPs that drive GTP hydrolysis in Arf1, remain unresolved owing to their overlapping basic function, endowed by the highly conserved ArfGAP domain (Poon et al., 1999). Recent structural models of COPI, based on cryo-electron tomography (Bykov et al., 2017; Dodonova et al., 2017), have shed light on the complex interplay of proteins involved in the COPI vesicle cycle and now offer structurally motivated hypotheses to resolve these issues. Harnessing recent structural information in an *in vivo* dissection, we now demonstrate that the activity of both ArfGAPs and the subsequent GTP hydrolysis in Arf1 triggers distinct cellular processes, despite the fact that their basic ArfGAP activities can substitute each other – because yeast strains lacking one or the other can survive. Our dissection pinpoints key differences between ArfGAPs and their spatially segregated regulation of Arf1. We also identify a so-far-unknown phospho-regulatory mechanism that potentially serves as a molecular timer on the ArfGAP-controlling basic aspects of COPI coat turnover. We provide a model that solves the conundrum of the seemingly redundant functions of ArfGAPs. Furthermore, we assign roles to each ArfGAP, which fit the molecular environment in which they exist in COPI.

## RESULTS

### Glo3 – not Gcs1 – is stably associated with COPI

The two ArfGAPs, Glo3 (ArfGAP2/3) and Gcs1 (ArfGAP1) regulate COPI function in yeast (Poon et al., 1999). Early studies have implicated ArfGAP1 as the relevant GAP and proposed ArfGAP1 as a functional component of the coat (Yang et al., 2002). Others have demonstrated that COPI also associates with Glo3/ArfGAP2/3 (Frigerio et al., 2007; Lewis et al., 2004). Elucidation of the structure of the COPI coat on reconstituted vesicles (Dodonova et al., 2017, 2015) and insights into the molecular interactions of either ArfGAP with coatomer (Rawet et al., 2010; Schindler et al., 2009; Suckling et al., 2014; Watson et al., 2004) lead to the hypothesis that they exist in contrasting molecular environments (Fig. 1A), just like the two Arf1 GTPase molecules associated with the coat (Dodonova et al., 2017). Interestingly, this hypothesis and a structure encompassing the ArfGAP domain of Glo3/ArfGAP2/3 (Dodonova et al., 2017) predicts that this ArfGAP occupies a niche where three Glo3/ArfGAP2/3 molecules come into close proximity at the centre of a COPI triad (Fig. 1B).

On the basis of the different binding parameters regarding the molecular interactions underlying this hypothesis (Pevzner et al., 2012; Suckling et al., 2014; Watson et al., 2004), we expected to observe differences in how stably the two yeast ArfGAPs interact with COPI and tested this hypothesis by affinity purification. Enrichment of GFP-tagged Glo3 or Gcs1 from cytosol (Fig. 1C) or from detergent extracts (Fig. 1D,E) revealed that, in contrast to Gcs1, COPI is strongly associated with Glo3, the yeast homologue of ArfGAP2/3. A number of other complexes that interact with COPI, such as Erv41–Erv46 (Rhiel et al., 2018 preprint; Shibuya et al., 2015), the yeast homologue of the mammalian ERGIC2–ERGIC3 complex, specifically co-purified with Glo3 (Fig. 1E, Table S3). This suggests that COPI and Glo3 – but not COPI and Gcs1 – are in a stable complex.

Glo3 binds COPI through the appendage domain of the γ-COP subunit (Pevzner et al., 2012; Watson et al., 2004) and occupies a niche adjacent to Arf1 created by the polymerising coat (Dodonova et al., 2017). Gcs1 binds the μ-homology domain (μHD) of δ-COP through a C-terminal δ-COP ligand (δL) tryptophan-based (WxxF) COPI recognition signal (Cosson et al., 1998; Rawet et al., 2010; Suckling et al., 2014). Binding affinities of the δ-COP μHD for peptides containing the tryptophan-based motif were reported to be in the low micromolar range (Suckling et al., 2014), possibly explaining why the COPI coat does not co-purify with Gcs1 (Fig. 1C-E).

### The stable association between Glo3 and COPI maps to a unique domain within Glo3

**Fig. 2.**
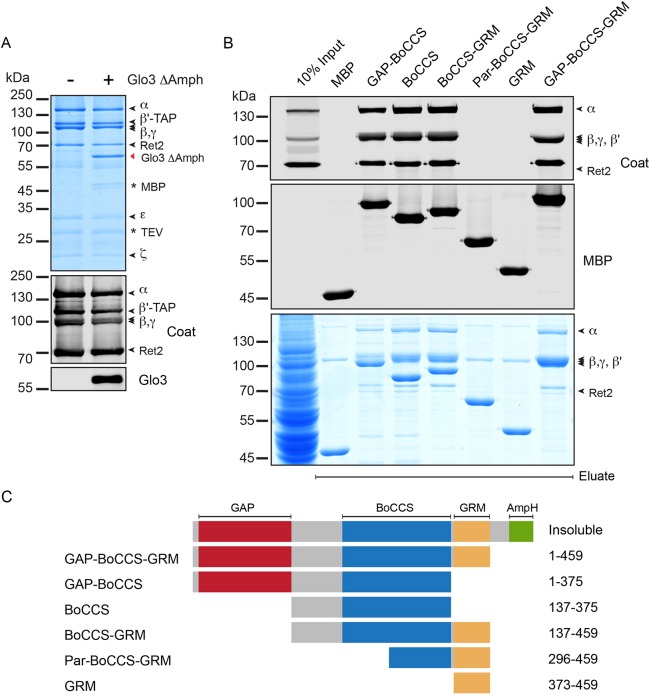
**Glo3 forms a stoichiometric complex with COPI *in vitro*.** (A) *In vitro* reconstitution of a COPI-Glo3 complex using TAP-purified coatomer, and recombinantly expressed and purified Glo3 lacking its distal amphipathic helix (459–493). Eluates obtained by Tobacco-etch virus (TEV) protease elution of TAP-tagged β′-COP after incubation with or without Glo3 (1–459) were analysed by Coomassie Blue staining of SDS-PAGE gels (top) or analysed by the immunodetection of western blots (bottom) using a coat antiserum detecting five of the seven subunits, or a Glo3 antibody. (B) Binding of coatomer to MBP fusion proteins of Glo3 from yeast lysates. The bound fraction was eluted and analysed by SDS-PAGE. Western blots were detected using a coat antiserum recognising five of the seven coatomer subunits. (C) Schematic illustration of the MBP-tagged truncations of Glo3. GAP, GTPase-activating domain; BoCCS, Binding of Coatomer, Cargo and SNARE domain; GRM, Glo3 regulatory motif; AmpH, amphipathic helix.

To test whether the stable association between Glo3 and COPI is due to direct binding, we reconstituted the interaction from purified components. We recombinantly expressed Glo3 lacking a C-terminal amphipathic helix to aid protein solubility, and found it to associate stoichiometrically with coatomer purified from *S. cerevisiae* (Fig. 2A).

Truncations of Glo3 were designed to reassess which Glo3 domains are necessary and sufficient for the stable association of Glo3 and COPI (Schindler et al., 2009). Binding experiments with partial constructs of Glo3 containing the GAP domain and the Binding of Coatomer, Cargo and SNARE (BoCCS) domain, the BoCCS domain alone (aa 137–375), the BoCCS domain and the evolutionarily conserved Glo3 regulatory motif (GRM), or the GRM alone clearly revealed that the BoCCS domain of Glo3 is crucial to bind coatomer directly (Fig. 2B,C). A construct (aa 296–459) lacking a stretch of positively charged residues in the BoCCS domain, attributed to mediate COPI binding (Kliouchnikov et al., 2008; Schindler et al., 2009), did not bind COPI. Similarly, a charge reversal of the same positively charged residues in the Glo3 BoCCS domain to negatively charged glutamic acid (E) residues, resulted in loss of COPI binding (Fig. S1A). Glo3 and its mammalian homologues contain a highly conserved tandem repeat of an ISSxxxFG sequence (Yahara et al., 2006) and this GRM regulates the function of Glo3 (Schindler et al., 2009). Our binding data show that the GRM alone is not sufficient to confer a stable interaction between Glo3 and COPI. More generally, the direct and robust association of COPI and Glo3 raises the possibility that Glo3 fulfills an additional function beyond its recognised role in GTP hydrolysis.

### GAP action on β- and γ-Arf1 is functionally distinct

The intrinsic GTPase activity of Arf1 is negligible and requires an ArfGAP to catalyse GTP hydrolysis (Kahn and Gilman, 1986; Randazzo and Kahn, 1994). Neither ArfGAP is essential in yeast or mammals, implying a certain degree of functional redundancy. However, the combined deletion of both is lethal (Frigerio et al., 2007; Poon et al., 1999).

Arf1 binds virtually equivalent sites on the homologous β- and γ-COP subunits (Yu et al., 2012). However, in the recently elucidated structural context of the membrane-associated COPI coat, β- and γ-Arf1 (denominated by the bound COPI subunit) occupy structurally distinct molecular environments within the coat lattice (Dodonova et al., 2017) (Fig. 1A,B). The GAP domain of ArfGAP2 has been observed to be positioned only near γ-Arf1 and not near β-Arf1 (Dodonova et al., 2017). Taking into consideration the positional restrictions imposed by the flexible, interacting domains of δ- and γ-COP, it is tempting to speculate that Gcs1/ArfGAP1 is positioned near β-Arf1.

It remains unclear how the respective position of either Arf1 molecule and, by extension, of either ArfGAP affects their function in the COPI vesicle cycle. The different molecular environments as well as the presence of non-conserved C-terminal domains in the ArfGAPs suggest distinct functions that currently are mechanistically unexplored.

**Fig. 3.**
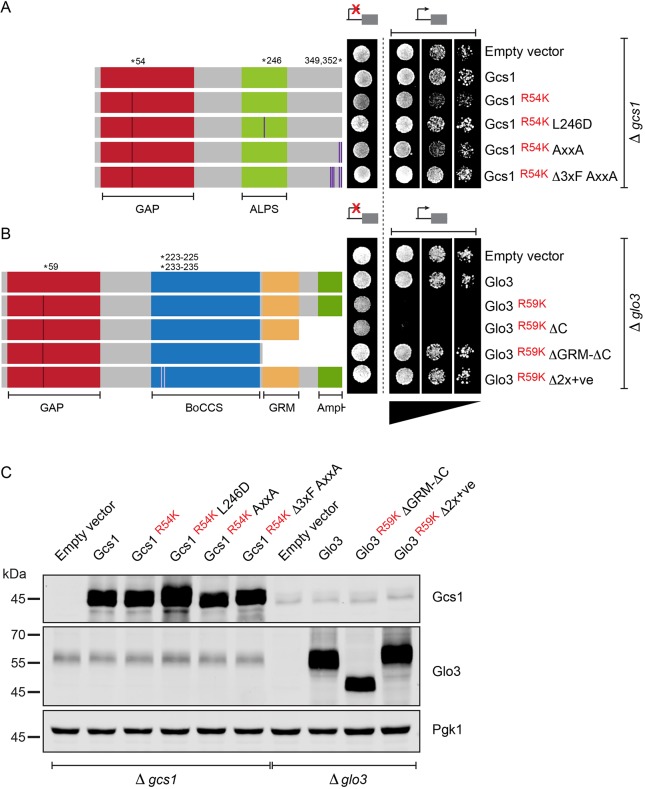
**Gcs1 and Glo3 regulate distinct cellular functions.** (A) Growth assay of Δ*gcs1* strains harbouring the indicated constructs on synthetic dropout medium analysed under *MET25*-promoter-repressing conditions (high methionine; control; indicated by a repressed promoter) or *MET25*-promoter-inducing conditions (normal methionine; test; indicated by an active promoter). A GAP-dead mutant of Gcs1 (Gcs1 ^R54K^) supports growth at 30°C. Gcs1 L246D, ALPS mutant; Gcs1 AxxA, alanine for tryptophan substitution of the C-terminal tryptophan-based COPI recognition signal; Gcs1 Δ3xF AxxA, alanine substitution of the C-terminal tryptophan-based COPI recognition signal and three upstream phenylalanine residues. GAP, GTPase-activating domain; ALPS, ArfGAP1 lipid-packing sensor. (B) Growth assay of Δ*glo3* strains harbouring the indicated constructs on synthetic dropout medium was assayed under *MET25* promoter-repressing conditions (high methionine; Control; indicated by a repressed promoter) and *MET25*-promoter-inducing conditions (normal methionine; test; indicated by an active promoter). A GAP-dead mutant of Glo3 (Glo3 ^R59K^) does not support growth at 30°C, indicating that GTP hydrolysis stimulated by Glo3 in γ-Arf1 is essential. Glo3 ΔC, deletion of the amphipathic helix; Glo3 ΔGRM-ΔC, combined deletion of the amphipathic helix and the GRM; Glo3 Δ2x+ve, alanine substitution for the COPI-binding region. GAP, GTPase-activating domain; BoCCS, Binding of Coatomer, Cargo and SNARE domain; GRM, Glo3 regulatory motif; AmpH, amphipathic helix. (C) Expression analysis of proteins in the indicated strains grown under *Met25*-promoter-inducing conditions (normal methionine; test).

To unravel these distinct functions, we first sought to inhibit the domain that is most conserved within Glo3 and Gcs1. The GAP domain of Glo3 and Gcs1 contains a highly conserved arginine residue (R54 in Gcs1 and R59 in Glo3). This arginine finger is essential for GAP function. Mutation of the arginine to a lysine dramatically impairs the GAP activity of both Gcs1 and Glo3 (Lewis et al., 2004; Yanagisawa et al., 2002). These dominant-negative forms of either ArfGAP fill their respective niches unproductively, without stimulating GTP hydrolysis in Arf1. Expression of the GAP-dead mutants in strains of their respective deletion backgrounds (i.e. expression of Glo3 R59K in Δ*glo3* and Gcs1 R54K in Δ*gcs1* strains) resulted in remarkably distinct phenotypes (Fig. 3). Cells harbouring the Gcs1 GAP-dead mutant were viable (Fig. 3A) while expression of the GAP-dead Glo3 caused lethality (Fig. 3B). All proteins were expressed to high steady-state protein levels (Fig. 3C), which excludes the lack of expression as a trivial explanation for the ability of the cells to tolerate the GAP-dead Gcs1.

Only a minor decrease in growth rate was observed in GAP-dead Gcs1-expressing cells. To test whether this effect was COPI-related we compared variants of GAP-dead Gcs1 that affected membrane recruitment or COPI interaction. We introduced a point mutation in the ALPS motif (L246D), which disrupts it and impairs the recruitment of Gcs1 to the membrane (Bigay et al., 2005; Xu et al., 2013). Similarly, we introduced a substitution combining the replacement of the C-terminal tryptophan-based motif (Rawet et al., 2010; Suckling et al., 2014) and three upstream phenylalanine residues – a manipulation expected to affect the binding of Gcs1 to δ-COP and to impair the targeting to its putative niche in the COPI coat. Both variants appeared to rescue the minor decline in growth (Fig. 3A).

We also investigated the molecular determinants of the toxic effects exerted by GAP-dead Glo3. The lethality caused by the expression of GAP-dead Glo3 was reversed by either the deletion of the GRM or the mutation of a stretch of positively charged residues (Δ2x+ve) in the BoCCS domain, previously ascribed to coordinate COPI binding (Kliouchnikov et al., 2008; Schindler et al., 2009), which is consistent with the direct binding data presented in Fig. 2 and Fig. S1A. Importantly, those GAP-dead variants that did not negatively affect growth were also expressed to high steady-state protein levels (Fig. 3C). In contrast, deletion of the conserved C-terminal amphipathic motif of Glo3, which governs the Golgi-localisation of ArfGAP3 (Kliouchnikov et al., 2008), was unable to reverse the dominant-negative lethality induced by GAP-dead Glo3 (Fig. 3B). Deletion of the C-terminal amphipathic helix of Glo3 did neither cause a change in its steady-state subcellular localisation nor its association with COPI (Fig. S2). On the basis of its strongly reduced association with COPI (Fig. S1), the COPI-binding deficient mutant (Δ2x+ve) fails to target GAP-dead Glo3 to the COPI coat, phenocopying Δ*glo3*. Cell viability upon deletion of GRM in the GAP-dead mutant could either indicate a failed targeting of Glo3 to COPI or report on the influence of an intact GRM in executing GAP-domain function. The interaction between Glo3 ΔGRM and COPI was unaltered (Fig. 2B, Fig. S2A), in favour of the latter hypothesis.

This phenotypic difference in viability caused by the expression of either GAP-dead mutant indicates that locking γ-Arf1 in its GTP-bound state through catalytically inactive Glo3 – but not β-Arf1 through catalytically inactive Gcs1 – is detrimental to the cell, in turn implying that GTP hydrolysis in either Arf1 triggers mechanistically distinct outcomes.

### Snf1/AMPK phosphorylates the GRM

**Fig. 4.**
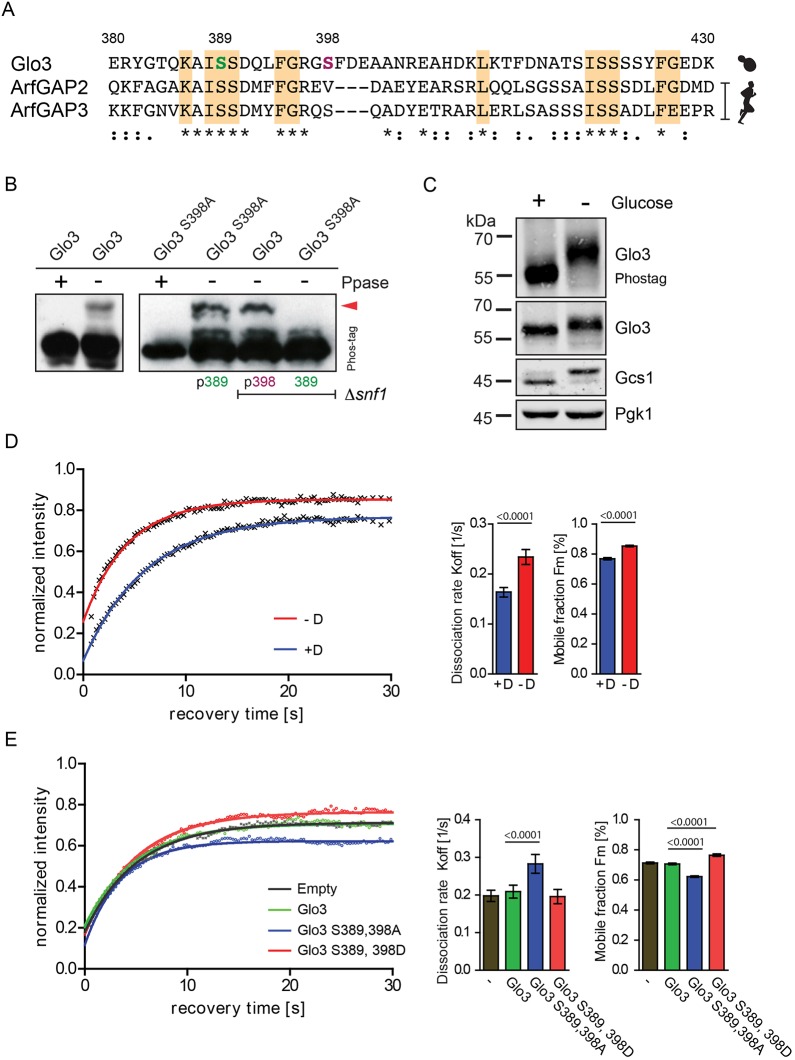
**Snf1 phosphorylates Glo3 at S389.** (A) Sequence alignment of the GRM domain of *S. cerevisiae* Glo3, and human ArfGAP2 and ArfGAP3. Asterisks indicate fully conserved residues; colons and full stops, respectively, indicate residues that are either strongly or weakly conserved. Regions of highest conservation – in comparison with other eukaryotes – are highlighted in orange. Residues S389 and S398 are indicated in green and purple, respectively. (B) Δ*glo3* strains expressing the indicated constructs under the control of a Tef1 promoter were analysed by Phos-tag PAGE. The electrophoretic mobility shift (arrowhead) indicates that residues S389 and S398 are phosphorylated in the presence and absence, respectively, of the Snf1 kinase. Treatment with λ-phosphatase (Ppase) resulted in dephosphorylation. (C) Analysis of proteins by Phos-tag PAGE and SDS-PAGE. Cells in mid-logarithmic phase were grown in the presence of glucose (+) or glucose starved (−) for 3 h prior to lysis. Western blots were immunodetected with the indicated antibodies. (D) Arf1-GFP fluorescence recovery after photobleaching (FRAP) in the presence (+D) or absence (−D) of glucose. Cells were grown to mid-logarithmic phase and glucose starved for 2 h prior to FRAP measurement. The plot, reflecting the recovery of Arf1-GFP at Golgi membranes (Vrg4-mCherry), shows the mean FRAP curves with the fits. The bar graphs on the right show *K*_off_ values per second (left) and Fm values in percent (right). (E) Arf1-GFP fluorescence recovery after photobleaching (FRAP) analysis in Δ*glo3* strains expressing the indicated Glo3 phospho-mutants. The plot, reflecting the recovery of Arf1-GFP at Golgi membranes (Vrg4-mCherry), shows the mean FRAP curves with the fits. The bar graphs on the right show *K*_off_ values per second (left) and Fm values in percent (right).

Since it is the BoCCS domain and not the GRM that is responsible for the stable interaction between Glo3 and COPI, we next explored determinants of toxicity within the GRM of GAP-dead Glo3 (Fig. 4A). The GRM contains four predicted phosphorylation sites (S389, S398, S423 and S424) (Blom et al., 1999). Sequential mutation of these residues identified two phosphosites within the GRM, S389 and S398 (Fig. 4B and Fig. S3). Affinity purification following stabilising crosslinking led to the identification of *S. cerevisiae* Snf1 as the kinase phosphorylating S389 (Fig. 4B and Fig. S3A-E). The Snf1/AMP-activated protein kinase (AMPK) is a key sensor of cellular energy levels and plays a vital role in the subsequent adaptation to metabolic stress (Hardie et al., 1998). Indeed, Glo3 appeared heavily phosphorylated when cells were starved for glucose (Fig. 4C), a condition that strongly activates Snf1.

To test whether this condition and, hence, Snf1 activation affects the presence of Arf1 on Golgi membranes, we used fluorescence recovery after photobleaching (FRAP) (Huranova et al., 2016) to investigate the dissociation kinetics of Arf1-GFP from Golgi membranes, as identified by the polytopic membrane protein Vrg4-mCherry (Fig. S4; Fig. 4D). Interestingly, glucose starvation increased dissociation of Arf1 from these membranes (*K*_off_ 0.234 s^−1^ vs 0.164 s^−1^) and decreased the immobile fraction of Arf1-GFP (Table S4). Mutation of serine residues 389 and 398 to aspartic acid (D) in Glo3, yielded a phosphomimetic Glo3 mutant (S389, 398D) designed to simulate glucose starvation; its expression similarly increased the dissociation of Arf1 from Golgi membranes (Fig. 4E and Table S4). By contrast, expression of an S389A and S398A Glo3 double mutant (S389, 398A) led to a decrease in the dissociation of Arf1 from Golgi membranes. These results support the notion that COPI-dependent vesicular traffic is less active under conditions of glucose starvation, when the GRM of Glo3 is phosphorylated. We conclude that phosphorylation of Glo3 by Snf1 contributes to this regulation. In fact, Snf1 has previously been implicated in phosphorylating a number of proteins involved in vesicle trafficking, including Gcs1, Glo3 and Age2 (Braun et al., 2014). Also, a study in HeLa cells has demonstrated that AMPK plays a crucial role in inhibiting vesicular transport pathways upon energy and nutrient starvation (Yang et al., 2018). Consistent with this study, we demonstrate that, in addition to Glo3, the other ArfGAP Gcs1 also appears to be post-translationally modified upon glucose starvation – an observation based upon the electrophoretic migratory differences following SDS-PAGE (Fig. 4C). However, for Glo3 a second serine residue, i.e. S398, was phosphorylated in the absence of Snf1, implicating another not-yet-identified kinase acting on this ArfGAP (Fig. 4B, Fig. S3C). These findings suggest that regulation of Glo3 through phosphorylation of the GRM is complex.

### Phosphorylation of the GRM regulates Glo3 function

**Fig. 5.**
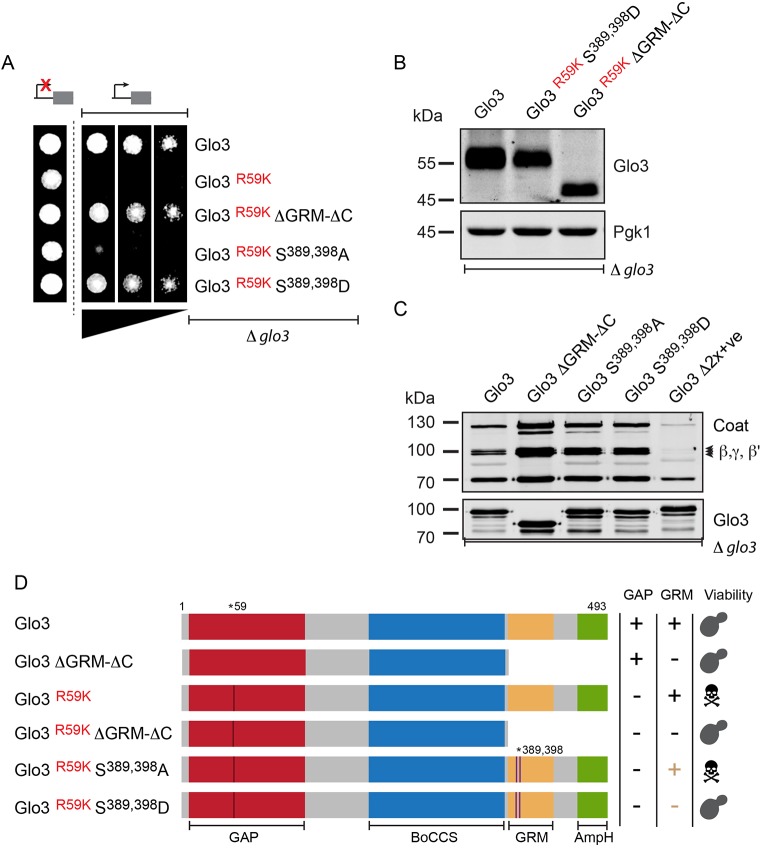
**Phosphorylation and/or dephosphorylation regulates Glo3 function.** (A) Growth assay of Δ*glo3* strains harbouring the indicated constructs on synthetic dropout medium analysed under *MET25*-promoter-repressing conditions (high methionine; control; indicated by a repressed promoter) and *MET25*-promoter-inducing conditions (normal methionine; test; indicated by an active promoter). The phosphomimetic mutant of the Glo3 motif rescues the dominant-negative effect of the GAP-dead Glo3 indicating that phosphorylation and/or dephosphorylation of the Glo3 motif regulates Glo3 function. (B) Expression analysis of proteins in the indicated strains grown under *Met25*-promoter-inducing conditions (normal methionine; test). (C) Affinity chromatography of GFP-tagged Glo3 variants from detergent extracts of Δ*glo3* strains harbouring the indicated GFP-tagged constructs and subsequent evaluation of COPI association. Glo3 ΔGRM-ΔC: combined deletion of amphipathic helix and Glo3 motif; Glo3 S389, 398A: alanine substitution for the indicated serines (non-phosphorylated mimetic); Glo3 S389, 398D: aspartic acid substitution for the indicated serine residues (phosphomimetic). Glo3 Δ2x+ve: alanine substitution for the COPI-binding region. (D) Summary. The Glo3 motif regulates the function of the GAP domain. Inactivation of the Glo3 motif by truncation or phosphorylation (phosphomimetic) rescues the dominant-negative lethality caused by a non-functional GAP domain. Inferred functionality of the GRM domain is highlighted in brown. GAP, GTPase activating domain; BoCCS, Binding of Coatomer, Cargo and SNARE domain; GRM, Glo3 regulatory motif; AmpH, amphipathic helix.

We hypothesised that regulation through the identified phosphosites converges on the same crucial function that was revealed by the fact that the GRM is required to render a GAP-dead Glo3 toxic (Fig. 3B). Therefore, we combined the Glo3 (S389, 398D) and the Glo3 (S389, 398A) mutants with the catalytically inactive R59K substitution (Fig. 5A). Expression of the non-phosphorylated GAP-dead mutant caused lethality, whereas the phosphomimetic mutant counteracts the dominant-negative lethality caused by disabling the GAP domain, supporting growth (Fig. 5A). All proteins were expressed to high steady-state protein levels (Fig. 5B). Thus, phosphorylation of the GRM, like deletion of the entire GRM, rescued the toxicity of the GAP-dead mutant, indicating that phosphorylation inactivates the GRM.

Next, we tested the possibility whether Glo3 binding of coatomer can also be regulated by phosphorylation of the GRM. In contrast to the mutation of a stretch of positively charged residues coordinating COPI binding through the BoCSS domain (Fig. 3B and Fig. S1A), which led to a loss of coatomer association with Glo3, both phospho-site variants and truncated Glo3 that lacks its GRM, were found to be associated with COPI to a similar extent (Fig. 5C). These results rule out defective Glo3-to-COPI targeting as the basis of viability in the presence of the phosphomimetic mutations in the GRM (Fig. 5A). Instead, our findings implicate regulation of GRM as a determinant of viability in cells that express a catalytically inactive form of Glo3 (Fig. 5D). This would indicate that, in the presence of a functional GRM, GTP hydrolysis in γ-Arf1 is essential. Moreover, it suggests that the GRM senses conformational changes triggered by GTP-hydrolysis and that its phosphorylation/dephosphorylation status impinges on its functionality. In line with this conclusion, substitution of S389 to either alanine (constitutively non-phosphorylated) or aspartic acid (phosphomimetic) precluded the ability of wild-type Glo3 to suppress the growth phenotype of a COPI hypomorph (*sec26^FW^;* F856A, W860A) (Fig. S3F). This result suggests that robust COPI function under different growth conditions relies on the regulation of Glo3 through phosphorylation and dephosphorylation – as expected for a reversible post-translational modification.

### Cargo sorting occurs in the molecular environment of β-Arf1

**Fig. 6.**
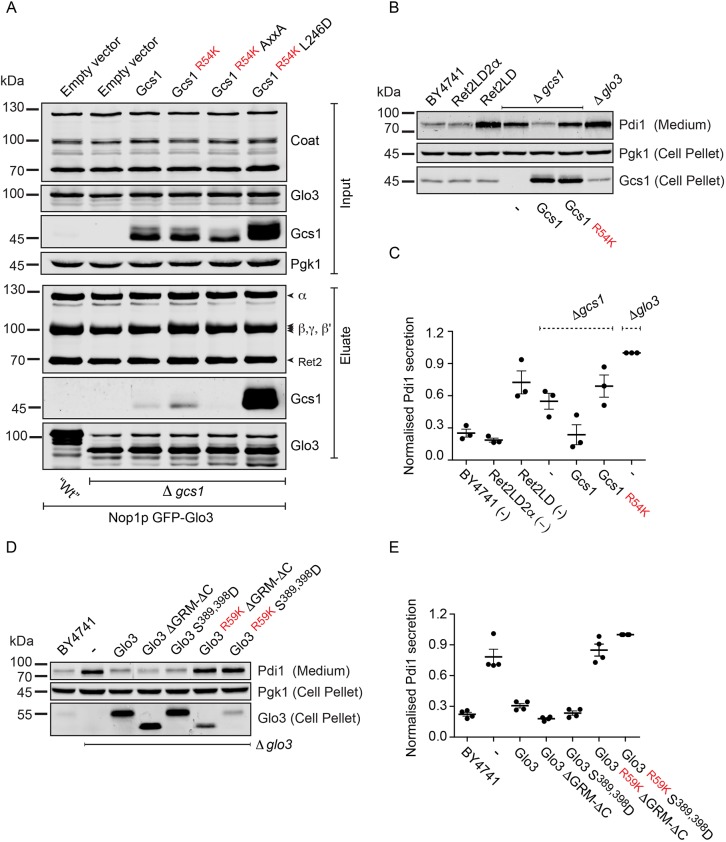
**Manipulation of Gcs1 or COPI in the β-Arf niche has similar effects on cargo sorting.** (A) Affinity chromatography of GFP-tagged Glo3 from detergent extracts of Δ*gcs1* strains harbouring the indicated Gcs1 constructs and subsequent evaluation of COPI and Gcs1 association. Gcs1 R54K, GAP-dead mutant of Gcs1; L246D, ALPS mutant; Gcs1 AxxA, alanine for tryptophan substitution of the C-terminal tryptophan-based COPI recognition signal. (B) Secretion assay of the indicated strains harbouring the indicated plasmids. Proteins secreted into the culture medium were analysed by SDS-PAGE and immunoblot analysis using antibodies specific for Pdi1. Cell pellets from the same cultures were also analysed using antibodies specific for Pgk1 and Gcs1. (C) Plot of the relative amounts of Pdi1 secreted into the culture medium by the indicated strains, as quantified by the densiometric analysis of immunoblots. Quantification of three independent experiments. Error bars depict ±s.e.m. (D) Secretion assay of the indicated strains harbouring the indicated plasmids. Proteins secreted into the culture medium were analysed by SDS-PAGE and immunoblot analysis using antibodies specific for Pdi1. Cell pellets from the same cultures were also analysed using antibodies specific for Pgk1 and Glo3. (E) Plot of the relative amounts of Pdi1 secreted into the culture medium by the indicated strains, as quantified by the densiometric analysis of immunoblots. Quantification of four independent experiments. Error bars indicate±s.e.m.

Deletion of either Glo3 or Gcs1 is tolerated by yeast cells, indicating that the presence of one of the GAPs is sufficient to mediate COPI-dependent transport. Therefore, the question arises whether the model that COPI associates with both ArfGAPs simultaneously is correct. To test the model, we enriched Glo3-associated coat and probed for the presence of Gcs1 (Fig. 6A). Consistent with the low affinity of Gcs1 to the μHD of δ-COP (Suckling et al., 2014), no endogenous Gcs1 co-purified with Glo3. When overexpressed, Gcs1 weakly co-precipitated with Glo3 and COPI. The overexpressed GAP-dead R54K mutant appeared to associate more stably with the coat (Fig. 6A). This association was lost upon mutation of the tryptophan-based motif present at the C-terminus of Gcs1. Curiously, mutation of the ALPS motif strongly increased co-purification of Gcs1 with Glo3 (presumably through COPI). This stabilisation possibly reflects a decrease in Gcs1 dissociation from COPI due to a loss of curvature sensing through the ALPS motif or could indicate that the ALPS motif masks the tryptophan-based motif in solution. We conclude that COPI can bind Glo3 and Gcs1 simultaneously, and that this *in vitro* transient complex between Glo3/ArfGAP2/3, coatomer and Gcs1/ArfGAP1 can be stabilised by manipulating Gcs1/ArfGAP1. This, substantially, strengthens our hypothesis that the two different ArfGAPs, akin to the two Arf GTPases, occupy distinct molecular niches within the COPI coat (Fig. 1A,B; Dodonova et al., 2017; Suckling et al., 2014).

The niche harbouring β-Arf1 and, plausibly also Gcs1/ArfGAP1 (Figs 1A, 6A), plays an important role in the retrieval of HDEL/KDEL-containing proteins. In this region of COPI, the recently discovered helix-b of δ-COP (Arakel et al., 2016; Dodonova et al., 2017) binds the switch and interswitch of β-Arf1, a region in Arf1 that is occluded by the N_O_ amphipathic helix in its inactive state (Dodonova et al., 2017). Given its ascribed role in cargo sorting (Arakel et al., 2016), its association with Arf1 (Dodonova et al., 2017) and the recognised coupling of Arf1 GTP hydrolysis to cargo sorting (Lanoix et al., 1999; Nickel et al., 1998), it is plausible that the helix translates GTP hydrolysis in β-Arf1 to COPI.

Perturbation of genes encoding for machinery involved in retrograde trafficking results in the secretion of soluble luminal ER residents, such as chaperones, bearing HDEL (KDEL in higher eukaryotes) sequences (Aguilera-Romero et al., 2008; Belden and Barlowe, 2001). Secretion of HDEL-containing proteins can occur either owing to inefficient retrieval of the HDEL receptor (Erd2) or activation of the unfolded protein response (UPR) during which the capacity of the HDEL-retrieval pathway is overwhelmed by the elevated expression of HDEL-bearing chaperones. Both situations are entwined and not mutually exclusive.

Deletion of Gcs1 leads to the secretion of HDEL-bearing protein disulfide-isomerase (Pdi1; Fig. 6B,C) and the induction of UPR (Jonikas et al., 2009). Deletion of δ-COP helix-b, which contacts β-Arf1, also results in the secretion of Pdi1 (Fig. 6B,C). Expression of functional Gcs1, but not GAP-dead Gcs1, rescues the observed secretion phenotype in a Gcs1-deletion strain, implying that GAP-stimulated GTP hydrolysis regulates HDEL retrieval. The upstream role of this specific ArfGAP and the downstream role of the δ-COP helix-b in the retrieval of the HDEL-bearing Pdi1 imply that δ-COP may relay the GTP hydrolysis in β-Arf1 to COPI.

Deletion of Glo3 also leads to the secretion of HDEL-bearing Pdi1 (Fig. 6B-E) and the induction of UPR (Jonikas et al., 2009). Expression of Glo3 rescues the observed phenotype in a Glo3-deletion strain (Fig. 6D,E). However, expression of the only two viable GAP-dead (R59K) mutants, the GRM deletion mutant (Glo3 ΔGRM-ΔC) and the S389, 398D phosphomimetic mutant, did not rescue the secretion phenotype. By contrast, expression of the same mutants in combination with a functional GAP domain led to complete rescue of the secretion phenotype (Fig. 6D,E). This suggests that the GRM of Glo3 is not crucial in HDEL-cargo sorting, whereas the GAP-domains of both Glo3 and Gcs1 are, indeed, crucial for efficient retrieval.

## DISCUSSION

Unlike the COPII vesicle coat, in which GAP activity is exerted through the Sec23 coat subunit (Yoshihisa et al., 1993), COPI relies on at least two accessory GAPs to stimulate GTPase activity in Arf1. In COPII, assembly of the inner Sec23–Sec24 coat complex with the outer cage (Sec13–Sec31) further stimulates GAP activity of Sec23 towards Sar1 (Antonny et al., 2001; Bi et al., 2007). Similar to COPII, COPI-associated ArfGAPs have higher activity when bound to Arf1 and COPI together (Goldberg, 1999; Pevzner et al., 2012). COPI and both ArfGAPs bind Arf1 non-competitively, unlike other GTPases that bind GAPs or their effectors in a competitive manner (Chen et al., 2012; Clabecq et al., 2000; Puertollano et al., 2001). This mechanism would require COPI-associated ArfGAPs to be subjected to regulation at multiple levels. ArfGAPs have also been implicated in cargo recognition and binding (Lee et al., 2005; Rein et al., 2002). Once bound, cargo regulates the activity of the ArfGAPs, potentially contributing to yet another tier of regulation (Goldberg, 1999; Luo et al., 2009). It is, therefore, not surprising that the precise orchestration of GTP-hydrolysis in Arf1 has remained a perplexing question in the COPI vesicle cycle.

ArfGAP1 activity is enhanced with increasing membrane curvature. This feature has been attributed to an amphipathic helix (ALPS) in Gcs1/ArfGAP1, which is unstructured in solution (Bigay et al., 2005, 2003). This helix senses lipid-packing defects, caused by the continual membrane curvature during vesicle formation, thereby determining the timing of catalytic activity in ArfGAP1. Like ArfGAP1, the activity of ArfGAP2 is enhanced in the presence of an intact coat (Goldberg, 1999; Luo et al., 2009; Pevzner et al., 2012; Szafer et al., 2001; Weimer et al., 2008). However, increasing membrane curvature does not enhance ArfGAP2 activity. The ArfGAP2 catalytic domain occupies a niche adjacent to γ-Arf1 that is formed only upon coat polymerisation, potentially providing one layer of regulation regarding its catalytic activity (Dodonova et al., 2017).

Owing to their overlapping functions, which may become apparent only under a strong selection pressure, presence of Gcs1 and Glo3 together is non-essential; however, the combined deletion of both is lethal (Poon et al., 1999). Cell viability is not compromised upon the defective targeting of either Gcs1 or Glo3 to COPI. Deletion of μHD (Arakel et al., 2016), mutation of the tryptophan-based COPI recognition motif in Gcs1 (Suckling et al., 2014), deletion of the γ-appendage domain or mutation of its COPI-binding site (Watson et al., 2004), or mutation of the COPI-binding region in Glo3 did not affect cell growth (Fig. 3B). However, by using GAP-dead mutants *in vivo*, we now established that cell viability is compromised when GAP-dead Glo3 occupies its niche. This is in marked contrast to GAP-dead Gcs1, where cell viability remains unaffected.

**Fig. 7.**
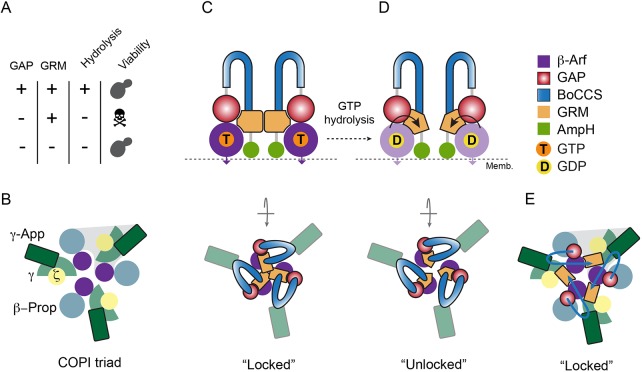
**The GRM domain regulates Glo3** **function.** (A) Summary of the key results obtained by manipulating the GAP and the GRM domain. (B) Schematic illustration of the COPI triad for structural orientation. (C) Adjacent GRM domains interconnect individual coat molecules at the heart of the COPI triad, effectively locking the triad together and stabilising COPI on the membrane. GTP hydrolysis in γ-Arf triggers a conformational change in the GRM domain and (D) uncouples adjacent GRM domains, effectively unlocking the triad and promoting the dissociation of COPI. (E) In this depiction, the GRM domain interconnects adjacent coat heptamers within a triad rather than adjacent GRM domains of neighbouring Glo3 molecules.

Inhibition of GTP hydrolysis in γ-Arf1 appears to abrogate the execution of a function essential for cell survival. Our findings reveal that cell viability is restored by impeding the function of the GRM either by its deletion or through its phosphorylation (phosphomimetic substitution), suggesting that GTP hydrolysis in γ-Arf1 culminates in the execution of a crucial function through the GRM (Fig. 7A). Conceivably, GTP hydrolysis in γ-Arf1 executes this crucial role through a conformational change, relayed either directly or indirectly to the GRM (Fig. 5D).

Inhibition of GTP hydrolysis by employing a GTP-locked constitutively active form of Arf1 comprising a Q71L point mutation (Arf1[Q71L]) or the use of non-hydrolysable analogues prevents membrane release of COPI (Presley et al., 2002; Tanigawa et al., 1993). Vesicle uncoating is a prerequisite of fusion and for the recycling of all coat components and occurs as a direct consequence of GAP-driven GTP hydrolysis. Given the importance of vesicle uncoating, the recycling of coat components and the crucial nature of the GTP hydrolysis in γ-Arf1, it is tempting to speculate that γ-Arf1, situated at the heart of a COPI triad (Dodonova et al., 2017), is primarily responsible for triggering coat disassembly.

We propose that, at the centre of the triad (Fig. 7B), Glo3 is tethered to the appendage domain of γ-COP and contacts other neighbouring Glo3 molecules or adjacent COPI heptamers of the triad through their GRM, effectively crosslinking individual coat molecules of the triad (Fig. 7C,E). GTP hydrolysis in γ-Arf1 and the subsequent conformational change in the GRM would disengage such a lock, freeing individual coat molecules for disassociation (Fig. 7D). In a mutant that lacks GAP activity this feedback would be lost, rendering individual coat molecules incapable of efficient disassembly from the triad and the membrane. Deletion of the GRM or electrostatic repulsion between its phosphorylated side chains possibly prevents the formation of such a lock, alleviating its adverse impact in the GAP-dead mutant. Moreover – or alternatively – phosphorylation may accelerate the unlocking of the COPI triad. In addition to the γ-appendage sandwich subdomain contacting β′-COP (Dodonova et al., 2017), a mechanism in which the GRMs interact homotypically (Fig. 7C) or crosslink individual COPI heptamers in the triad (Fig. 7E) may help further stabilise the COPI triad.

Such a model would help to clarify why overexpression of Glo3 with an intact GRM mitigates the temperature-sensitivity of mutants that harbour mutations in the appendage domain of β-COP or in Arf1 (De Regis et al., 2008; Yahara et al., 2006). The β-COP appendage serves as the main link between the cage-like (outer) and adaptor-like (inner) subcomplexes of the coat, (Dodonova et al., 2017), and mutations in it potentially destabilise the structure of the COPI coat and undermine its stability on membranes. Overexpression of Glo3 possibly ensures that its availability is not rate limiting and facilitates adequate buttressing of the triad (Fig. 7).

Phosphorylation and dephosphorylation of the GRM within Glo3 influences the dissociation of Arf1 from Golgi membranes (Fig. 4E). By influencing the dwell time of COPI on membranes, this model would also help explain both the ‘productive’ and ‘discard/futile’ states in the formation of COPI vesicles (Goldberg, 2000; Nie and Randazzo, 2006; Springer et al., 1999). The phospho-regulation of the possible stabilising effect through Snf1 might additionally serve as a kinetic timer that delineates productive from unproductive cycles or accelerates unlocking of the triad. Residue S432 in human ArfGAP2 and S431 in mice, corresponding to S389 in Glo3, was found to be phosphorylated in almost 50 high-throughput studies (www.phosphosite.org) suggesting that phosphorylation of the GRM by AMPK is evolutionarily conserved and physiologically relevant. Indeed, our finding fits other observations that suggest a regulation of retrograde traffic in the Golgi through AMPK (Miyamoto et al., 2008).

Our model also explains why the deletion of Glo3 leads to a secretion of HDEL-bearing Pdi1. Curtailing the dwell time of COPI on the membrane by deletion of Glo3 possibly affects the efficient sorting of cargo. In a similar manner, plasma-membrane-localised Snc1, whose recycling involves the first propeller domain of β′-COP (Xu et al., 2017), is mislocalised upon deletion of either Glo3 or Gcs1 (Kawada et al., 2015; Robinson et al., 2006). Although culminating in phenotypically similar outcomes, the underlying mechanism by which Gcs1 or Glo3 deletion causes HDEL-protein secretion is likely to be distinct.

Why have the two ArfGAPs evolved seemingly different affinities for the COPI coat? It is unknown whether Glo3/ArfGAP2 is associated with coatomer in the cytosol and recruited to membranes *en bloc*, or whether the detected stable association of COPI and Glo3 (Fig. 1) represents COPI triads undergoing dissociation in the cytosol.

β-Arf, which occupies a region of the coated vesicle whose membrane is more exposed (Dodonova et al., 2017), is conceivably more actively involved in cargo-sorting and release (Fig. 6). The δ-COP helix-b potentially relays the GTPase activity of β-Arf1 to COPI by functionally translating this signal into a cargo-binding and/or release directive. The region adjoining the longin domain of ζ-COP is also predicted to form an α-helix that is equivalent to the δ-COP helix-b (Alisaraie and Rouiller, 2012). It is unknown whether such a helix does exist or how GTP hydrolysis in γ-Arf1 is relayed to the coat. The pivotal role of GTP hydrolysis in γ-Arf1 raises the possibility that this did contribute to the evolutionary selective pressure underlying the gene duplication event in γ-COP, ζ-COP, ArfGAP2 and ArfGAP3; their alternative isoforms (Kliouchnikov et al., 2008; Moelleken et al., 2007) suggest additional plasticity of GTP hydrolysis in mammalian γ-Arf1, which may be modulated depending on the associated COPI isoform, and ArfGAP2 and ArfGAP3.

Although we can still only speculate about the precise mechanism by which COPI and ArfGAPs communicate, our data points to a model that reconciles the convoluted reports regarding the roles of Gcs1/ArfGAP1 and Glo3/ArfGAP2/3 in COPI vesicle biogenesis and uncoating.

## MATERIALS AND METHODS

### Yeast strains and plasmids

The CloNAT cassette was amplified from the pAG25 vector. The cassette was inserted in the *Saccharomyces cerevisiae* yeast genome by homologous recombination into the respective strains, deleting *GLO3* or *GCS1*. The knockout strains were verified by PCR from genomic DNA, using ORF-specific primers and antibodies against Glo3 and Gcs1. Yeast strains, plasmids and antibodies employed in this study are listed in Tables S1, S2, and S5 respectively

### Affinity chromatography

*S. cerevisiae* cells were grown to mid-logarithmic phase and harvested. The cell pellet was flash-frozen in liquid nitrogen, crushed by hand and resuspended in lysis buffer (25 mM Tris-HCl pH 7.4, 50 mM KCl, 10 mM MgCl_2_, 5% glycerol, 1% Triton X-100). Detergent was not added to the lysis buffer for the preparation of cytosolic extracts. After thawing, the samples were incubated on ice for 15 min. Cytosolic extracts were centrifuged twice at 150,000 ***g***. Detergent-solubilised extracts were centrifuged twice at 5,000 ***g***. The supernatant was incubated with Miltenyi µMACS GFP micro-beads for 30 min at 4°C. The affinity matrix and bound complexes were separated using Miltenyi µMACS isolation columns. The matrix was washed 4× with lysis buffer and eluted with SDS-sample buffer containing 100 mM DTT. The samples were either analysed by SDS-PAGE and western blotting or by liquid chromatography– tandem mass spectrometry (LC-MS/MS) analysis.

### Spotting assays

To control the expression, genes were placed downstream of the repressible *Met25* promoter. Yeast cells were transformed with the indicated plasmids and grown on synthetic complete medium lacking leucine (auxotrophic marker) and containing excess methionine (800 μM) to repress gene expression. Transformed cells were normalised (OD_600_) and spotted on low methionine-containing synthetic complete medium to induce gene expression. Cells were grown at 30°C for 3 days prior to analysis.

### *In vitro* reconstitution of the COPI-Glo3 complex

Rosetta (DE3) and BL21 (pREP4) *Escherichia coli* strains containing the MBP fusion constructs were induced by the addition of 0.2 mM IPTG at OD_600_ 0.8 for 3 h at 30°C. Cells were harvested and sonicated in lysis buffer [50 mM NaH_2_PO_4_, 300 mM NaCl, 15 mM imidazole, pH 8.0]. Lysates were cleared first by centrifugation at 5000 ***g*** for 5 min and, subsequently, at 100,000 ***g*** for 30 min. The MBP fusion proteins and baits were purified by incubating lysates with Ni-IDA resin. The affinity matrix was washed 5× with lysis buffer and eluted with elution buffer (50 mM NaH_2_PO_4_, 300 mM NaCl, 250 mM imidazole, pH 8.0). The baits were dialyzed overnight in dialysis buffer (200 mM NaCl, 20 mM HEPES, pH 7.4, and 1 mM DTT) before use in *in vitro* reconstitution assays.

Tandem affinity purification (TAP) of COPI was performed as described by Arakel et al., 2016. TAP-immobilised COPI was incubated with a molar excess of MBP-Glo3 (ΔAmpH) for 20 min at 4°C. The affinity matrix was washed 3× with lysis buffer and once with Tobacco etch virus protease (TEV) cleavage buffer (10 mM Tris-HCl, pH 8.0, 150 mM NaCl, 0.1% Triton X-100, 0.5 mM EDTA, and 1 mM DTT). The COPI-Glo3 complex was eluted by TEV cleavage for 1 h at 16°C. The eluates were incubated with amylose resin to deplete MBP.

### Fluorescence recovery after photobleaching, data processing and data analysis

The FRAP analysis was perfomed as described in Huranova et al., 2016. In brief, cells were grown in YPD medium overnight, diluted and grown for 4–6 h to mid-log phase in YPD or selective HC medium supplemented with adenine. Glucose starvation was carried out for 2 h prior to fluorescence recovery after photobleaching (FRAP) measurements. Cells were washed and mounted in HC complete or selective medium onto a 1.6% agarose pad either with or without glucose. FRAP measurements were performed using a Leica SP5-II-Matrix confocal microscope equipped with an oil immersion objective HCX Plan-Apochromat 63× NA 1.40-0.6 oil, Lbd Blue CS (Fig. 4D) or Leica TCS SP8 confocal microscope equipped with an oil immersion objective HC Plan-Apochromat 63× NA 1.4 oil, CS2 (Fig. 4E) at 27°C. Data acquisition was performed in 512×512 pixel format with pinhole 2.62 Airy, at a speed of 1000 Hz in bidirectional mode and 8-bits resolution. Photobleaching (0.3 s) was performed with a circular spot 1.0 µm in diameter using the 488 nm Ar line at 100% laser power. Fluorescence recovery was monitored at low laser intensity (2–5%) at 0.26 s intervals until reaching the plateau of recovery, in total for 43 s after photobleaching. A total of 25–30 separate FRAP measurements were performed for each sample. All FRAP curves were normalised to fluorescence loss during acquisition following subtraction of background fluorescence. Curve fitting was performed in GraphPad Prism software using the one-phase association fit, assuming the protein turnover at the cis-Golgi to be an elementary association and/or dissociation process. All individual curves were fitted at once to obtain the mean and 95% confidence intervals of the desired parameters, rate constant *K*_off_ and the mobile fraction F_m_.

### Secretion assay

Secretion assays were performed as described by Arakel et al., 2016.

### Statistics

All experiments were reproduced at least three times. The data were plotted as mean±s.e.m. Statistical significance (*P*<0.05) was assessed by a two-tailed *t*-test.

### Detection of Glo3 phosphorylation

Yeast strains expressing either Glo3-FLAG or variants thereof were grown to early-to-mid logarithmic phase in HC-Leu medium. The equivalent amount of cells from 50 OD_600_ units per strain were harvested and resuspended in 500 μl of lysis buffer [25 mM Tris-HCL pH7.5, 250 mM KCl, 100 mM glycine, 1 mM β-mercaptoethanol, 1 μM ZnCl_2_, 0.1% Triton X-100, 0.1 mM PMSF, protease inhibitor cocktail (Roche)]. An equal volume of glass beads was added and the cells were lysed using Fast-Prep (MP Biomedical; 4×15 s at 6.5), with 5-min intervals on ice. After lysis, phosphatase inhibitors (10×: 100 mM NaF, 100 mM NaN_3_, 100 mM pNPP, 100 mM NaPPi, 100 mM β-glycerophosphate) were added when needed. Unbroken cells and debris were removed by centrifugation for 2 min at 960 ***g*** and 4°C. The supernatant was incubated with 20 μl of M2 anti-FLAG beads (Sigma) per 3 mg of cell lysate for 2 h at 4°C. The beads were washed 3× in lysis buffer and 3× in wash buffer (25 mM Tris-HCl pH 8.0, 150 mM NaCl). The samples were analysed either by gel electrophoresis or mass spectrometry. When indicated, samples were treated with alkaline phosphatase for 1 h at 37°C prior to further analysis.

For gel analysis, samples were separated on 10% Phos-tag gels (Nard Technologies), transferred onto nitrocellulose and decorated with either a homemade or a commercially available anti-FLAG antibody, and developed by using a secondary anti-mouse-HRP antibody (Pierce 1:10,000) and ECL (GE Healthcare). For LC/MS/MS analysis, the beads were resuspended in 50 µl wash buffer. Protein digestion was started by adding 0.25 μg of ELC (Wako chemicals) and incubated for 2 h at 37°C. Digestion was continued with another 0.25 μg of ELC and 2 hr-incubation at 37°C. This was followed by a two-step digestion with trypsin (1×0.25 µg, 2 h, 37°C and 1×0.25 µg, overnight at 37°C). The peptides were collected by centrifuging the beads for 2 min at 420 ***g*** and the supernatant was acidified by the addition of 1/20 vol. of 10% trifluoroacetic acid. The samples were desalted using Microspin Vydac C18 silica columns according to the manufacturer's recommendation (The Nest group, Southborough, MA). The desalted peptides were dried and phosphopeptides were enriched with TiO_2_ according to Kettenbach and Gerber (2011). The phosphopeptide pool was desalted as above and analysed on an Orbitrap Elite instrument as described in Hindupur et al. (2018).

### Glo3 purification for kinase assay

Glo3, Glo3(214–493) [Glo3-H], and Glo3(214–493 S->A)[Glo3-H13] were expressed in a 1 l culture of *E. coli* BL21* as N-terminal His-SUMO fusion proteins. Bacterial pellets were lysed in 30 ml of 20 mM Tris-HCl pH 7.8, 200 mM KCl, 1% Tween 20 in the presence of 1 mg/ml lysozyme and protease inhibitor through sonication. The lysate was spun for 30′ at 24,000 ***g*** and 4°C. The supernatant was incubated with 0.4 g Protino Ni resin (Machery-Nagel) for 1 h at 4°C rotating end to end. The beads were washed 4× with 20 mM Tris-HCl pH 7.8, 200 mM KCl, 0.1% Tween 20, 2× with 20 mM Tris-HCl pH 7.8, 1 M KCl, 0.1% Tween 20 and again once with 20 mM Tris-HCl pH 7.8, 200 mM KCl, 0.1% Tween 20. Proteins were eluted with 250 mM imidazole in 20 mM Tris-HCl pH 7.8, 200 mM KCl, protein-containing fractions were pooled and dialyzed 3× against PBS 5% glycerol. The proteins were snap frozen in liquid N_2_ and stored at −80°C.

### Snf1 kinase assay

The Snf1 kinase assay is based on (Ruiz et al., 2012; Nicastro et al., 2015). Cells expressing Snf1-HA or the kinase mutant Snf1-K84R-HA or Δ*snf1* were collected by filtration, resuspended in SC complete medium (0.5% glucose) and incubated on a shaker at 30°C for 20 min, collected again by filtration and stored at −80°C. After cell lysis through vortexing in 50 mM Tris-HCl pH 7.5, 50 mM NaF, 5 mM Na^+^ pyrophosphate, 1 mM EDTA, 0.5% Triton X-100, 10% (vol/vol) glycerol and protease inhibitor cocktail (Pierce), the lysate was spun for 10 min at 13,000 ***g*** and 4°C; the supernatant was transferred to a fresh microfuge tube and spun for 5 min at 13,000 ***g*** and 4°C. The supernatant was incubated with 40 µl 50% slurry of anti-HA beads (Thermo Scientific) for 2 h at 4°C under end-to-end rotation. The beads were washed 3× with 50 mM Tris-HCl pH 7.5, 150 mM NaCl, 50 mM NaF, 1 mM EDTA, 1 mM DTT and protease inhibitors (Pierce), and 2× in 1× kinase buffer (20 mM HEPES pH 7.5, 100 mM NaCl, 0.5 mM EDTA, 5 mM MgAc_2_, 0.5 mM DTT). The IP efficiency was evaluated by immunoblotting. Snf1-HA or the kinase mutant were incubated with 6 µg of Glo3 or Glo3 variants in 1× kinase buffer and the presence of 100 µCi/ml ^32^P-ATP (SRP-301) and 1 mM ATP in a total reaction volume of 30 µl for 60 min at 30°C. Of the reaction, 13 µl was separated on a 4–15% SDS-PAGE gradient gel. The gel was stained with Coomassie Blue and a picture was taken before drying the gel on a filter paper for autoradiography.

## Supplementary Material

Supplementary information

## References

[JCS232124C1] Aguilera-Romero A., Kaminska J., Spang A., Riezman H. and Muñiz M. (2008). The yeast p24 complex is required for the formation of COPI retrograde transport vesicles from the Golgi apparatus. *J. Cell Biol.* 180, 713-720. 10.1083/jcb.200710025 18283113 PMC2265561

[JCS232124C2] Alisaraie L. and Rouiller I. (2012). Full-length structural model of RET3 and SEC21 in COPI: identification of binding sites on the appendage for accessory protein recruitment motifs. *J. Mol. Model.* 18, 3199-3212. 10.1007/s00894-011-1324-9 22246286 PMC3385859

[JCS232124C3] Aniento F., Gu F., Parton R. G. and Gruenberg J. (1996). An endosomal beta COP is involved in the pH-dependent formation of transport vesicles destined for late endosomes. *J. Cell Biol.* 133, 29-41. 10.1083/jcb.133.1.29 8601610 PMC2120778

[JCS232124C4] Antonny B., Beraud-Dufour S., Chardin P. and Chabre M. (1997). N-terminal hydrophobic residues of the G-protein ADP-ribosylation factor-1 insert into membrane phospholipids upon GDP to GTP exchange. *Biochemistry* 36, 4675-4684. 10.1021/bi962252b 9109679

[JCS232124C5] Antonny B., Madden D., Hamamoto S., Orci L. and Schekman R. (2001). Dynamics of the COPII coat with GTP and stable analogues. *Nat. Cell Biol.* 3, 531-537. 10.1038/35078500 11389436

[JCS232124C6] Arakel E. C., Richter K. P., Clancy A. and Schwappach B. (2016). δ-COP contains a helix C-terminal to its longin domain key to COPI dynamics and function. *Proc. Natl. Acad. Sci. USA* 113, 6916-6921. 10.1073/pnas.1603544113 27298352 PMC4922195

[JCS232124C7] Beck R., Rawet M., Ravet M., Wieland F. T. and Cassel D. (2009). The COPI system: molecular mechanisms and function. *FEBS Lett.* 583, 2701-2709. 10.1016/j.febslet.2009.07.032 19631211

[JCS232124C8] Belden W. J. and Barlowe C. (2001). Deletion of yeast p24 genes activates the unfolded protein response. *Mol. Biol. Cell* 12, 957-969. 10.1091/mbc.12.4.957 11294899 PMC32279

[JCS232124C9] Beller M., Sztalryd C., Southall N., Bell M., Jäckle H., Auld D. S. and Oliver B. (2008). COPI complex is a regulator of lipid homeostasis. *PLoS Biol.* 6, e292 10.1371/journal.pbio.0060292 19067489 PMC2586367

[JCS232124C10] Bi X., Mancias J. D. and Goldberg J. (2007). Insights into COPII coat nucleation from the structure of Sec23.Sar1 complexed with the active fragment of Sec31. *Dev. Cell* 13, 635-645. 10.1016/j.devcel.2007.10.006 17981133 PMC2686382

[JCS232124C11] Bigay J., Gounon P., Robineau S. and Antonny B. (2003). Lipid packing sensed by ArfGAP1 couples COPI coat disassembly to membrane bilayer curvature. *Nature* 426, 563-566. 10.1038/nature02108 14654841

[JCS232124C12] Bigay J., Casella J.-F., Drin G., Mesmin B. and Antonny B. (2005). ArfGAP1 responds to membrane curvature through the folding of a lipid packing sensor motif. *EMBO J.* 24, 2244-2253. 10.1038/sj.emboj.7600714 15944734 PMC1173154

[JCS232124C13] Blom N., Gammeltoft S. and Brunak S. (1999). Sequence and structure-based prediction of eukaryotic protein phosphorylation sites. *J. Mol. Biol.* 294, 1351-1362. 10.1006/jmbi.1999.3310 10600390

[JCS232124C14] Braun K. A., Vaga S., Dombek K. M., Fang F., Palmisano S., Aebersold R. and Young E. T. (2014). Phosphoproteomic analysis identifies proteins involved in transcription-coupled mRNA decay as targets of Snf1 signaling. *Sci. Signal.* 7, ra64-ra64 10.1126/scisignal.2005000 25005228

[JCS232124C15] Bykov Y. S., Schaffer M., Dodonova S. O., Albert S., Plitzko J. M., Baumeister W., Engel B. D. and Briggs J. A. (2017). The structure of the COPI coat determined within the cell. *Elife* 6, e32493 10.7554/eLife.32493 29148969 PMC5716667

[JCS232124C16] Chen K.-Y., Tsai P.-C., Liu Y.-W. and Lee F.-J. S. (2012). Competition between the golgin Imh1p and the GAP Gcs1p stabilizes activated Arl1p at the late-Golgi. *J. Cell Sci.* 125, 4586-4596. 10.1242/jcs.107797 22767516

[JCS232124C17] Clabecq A., Henry J.-P. and Darchen F. (2000). Biochemical characterization of Rab3-GTPase-activating protein reveals a mechanism similar to that of Ras-GAP. *J. Biol. Chem.* 275, 31786-31791. 10.1074/jbc.M003705200 10859313

[JCS232124C18] Cosson P., Lefkir Y., Démollière C. and Letourneur F. (1998). New COP1-binding motifs involved in ER retrieval. *EMBO J.* 17, 6863-6870. 10.1093/emboj/17.23.6863 9843492 PMC1171034

[JCS232124C19] De Regis C. J., Rahl P. B., Hoffman G. R., Cerione R. A. and Collins R. N. (2008). Mutational analysis of betaCOPI (Sec26p) identifies an appendage domain critical for function. *BMC Cell Biol.* 9, 3 10.1186/1471-2121-9-3 18211691 PMC2262067

[JCS232124C20] Dodonova S. O., Diestelkoetter-Bachert P., von Appen A., Hagen W. J. H., Beck R., Beck M., Wieland F. and Briggs J. A. G. (2015). A structure of the COPI coat and the role of coat proteins in membrane vesicle assembly. *Science* 349, 195-198. 10.1126/science.aab1121 26160949

[JCS232124C21] Dodonova S. O., Aderhold P., Kopp J., Ganeva I., Röhling S., Hagen W. J. H., Sinning I., Wieland F. and Briggs J. A. G. (2017). 9Å structure of the COPI coat reveals that the Arf1 GTPase occupies two contrasting molecular environments. *Elife* 6, e26691 10.7554/eLife.26691 28621666 PMC5482573

[JCS232124C22] Frigerio G., Grimsey N., Dale M., Majoul I. and Duden R. (2007). Two human ARFGAPs associated with COP-I-coated vesicles. *Traffic* 8, 1644-1655. 10.1111/j.1600-0854.2007.00631.x 17760859 PMC2171037

[JCS232124C23] Goldberg J. (1999). Structural and functional analysis of the ARF1-ARFGAP complex reveals a role for coatomer in GTP hydrolysis. *Cell* 96, 893-902. 10.1016/S0092-8674(00)80598-X 10102276

[JCS232124C24] Goldberg J. (2000). Decoding of sorting signals by coatomer through a GTPase switch in the COPI coat complex. *Cell* 100, 671-679. 10.1016/S0092-8674(00)80703-5 10761932

[JCS232124C25] Hara-Kuge S., Kuge O., Orci L., Amherdt M., Ravazzola M., Wieland F. T. and Rothman J. E. (1994). En bloc incorporation of coatomer subunits during the assembly of COP-coated vesicles. *J. Cell Biol.* 124, 883-892. 10.1083/jcb.124.6.883 8132710 PMC2119964

[JCS232124C26] Hardie D. G., Carling D. and Carlson M. (1998). The AMP-activated/SNF1 protein kinase subfamily: metabolic sensors of the eukaryotic cell? *Annu. Rev. Biochem.* 67, 821-855. 10.1146/annurev.biochem.67.1.821 9759505

[JCS232124C73] Hindupur S. K., Colombi M., Fuhs S. R., Matter M. S., Guri Y., Adam K., Cornu M., Piscuoglio S., Ng C. K. Y., Betz C. et al. (2018). The protein histidine phosphatase LHPP is a tumour suppressor. *Nature* 555, 678-682. 10.1038/nature26140 29562234 PMC6376988

[JCS232124C27] Huranova M., Muruganandam G., Weiss M. and Spang A. (2016). Dynamic assembly of the exomer secretory vesicle cargo adaptor subunits. *EMBO Rep.* 17, 202-219. 10.15252/embr.201540795 26742961 PMC5290816

[JCS232124C28] Jonikas M. C., Collins S. R., Denic V., Oh E., Quan E. M., Schmid V., Weibezahn J., Schwappach B., Walter P., Weissman J. S. et al. (2009). Comprehensive characterization of genes required for protein folding in the endoplasmic reticulum. *Science* 323, 1693-1697. 10.1126/science.1167983 19325107 PMC2877488

[JCS232124C29] Kahn R. A. and Gilman A. G. (1986). The protein cofactor necessary for ADP-ribosylation of Gs by cholera toxin is itself a GTP binding protein. *J. Biol. Chem.* 261, 7906-7911. 3086320

[JCS232124C30] Kawada D., Kobayashi H., Tomita T., Nakata E., Nagano M., Siekhaus D. E., Toshima J. Y. and Toshima J. (2015). The yeast Arf-GAP Glo3p is required for the endocytic recycling of cell surface proteins. *Biochim. Biophys. Acta* 1853, 144-156. 10.1016/j.bbamcr.2014.10.009 25409928

[JCS232124C72] Kettenbach A. N. and Gerber S. A. (2011). Rapid and reproducible single-stage phosphopeptide enrichment of complex peptide mixtures: application to general and phosphotyrosine-specific phosphoproteomics experiments. *Anal. Chem.* 83, 7635-7644. 10.1021/ac201894j 21899308 PMC3251014

[JCS232124C31] Kliouchnikov L., Bigay J., Mesmin B., Parnis A., Rawet M., Goldfeder N., Antonny B. and Cassel D. (2008). Discrete determinants in ArfGAP2/3 conferring golgi localization and regulation by the COPI coat. *Mol. Biol. Cell* 20, 859-869. 10.1091/mbc.e08-10-1010 19109418 PMC2633400

[JCS232124C32] Lanoix J., Ouwendijk J., Lin C. C., Stark A., Love H. D., Ostermann J. and Nilsson T. (1999). GTP hydrolysis by arf-1 mediates sorting and concentration of Golgi resident enzymes into functional COP I vesicles. *EMBO J.* 18, 4935-4948. 10.1093/emboj/18.18.4935 10487746 PMC1171565

[JCS232124C33] Lee S. Y., Yang J.-S., Hong W., Premont R. T. and Hsu V. W. (2005). ARFGAP1 plays a central role in coupling COPI cargo sorting with vesicle formation. *J. Cell Biol.* 168, 281-290. 10.1083/jcb.200404008 15657398 PMC2171589

[JCS232124C34] Lewis S. M., Poon P. P., Singer R. A., Johnston G. C. and Spang A. (2004). The ArfGAP Glo3 is required for the generation of COPI vesicles. *Mol. Biol. Cell* 15, 4064-4072. 10.1091/mbc.e04-04-0316 15254269 PMC515341

[JCS232124C35] Luo R., Ha V. L., Hayashi R. and Randazzo P. A. (2009). Arf GAP2 is positively regulated by coatomer and cargo. *Cell. Signal.* 21, 1169-1179. 10.1016/j.cellsig.2009.03.006 19296914 PMC2692659

[JCS232124C36] Miyamoto T., Oshiro N., Yoshino K.-I., Nakashima A., Eguchi S., Takahashi M., Ono Y., Kikkawa U. and Yonezawa K. (2008). AMP-activated protein kinase phosphorylates Golgi-specific brefeldin A resistance factor 1 at Thr1337 to induce disassembly of Golgi apparatus. *J. Biol. Chem.* 283, 4430-4438. 10.1074/jbc.M708296200 18063581

[JCS232124C37] Moelleken J., Malsam J., Betts M. J., Movafeghi A., Reckmann I., Meissner I., Hellwig A., Russell R. B., Söllner T., Brügger B. et al. (2007). Differential localization of coatomer complex isoforms within the Golgi apparatus. *Proc. Natl. Acad. Sci. USA* 104, 4425-4430. 10.1073/pnas.0611360104 17360540

[JCS232124C75] Nicastro R., Tripodi F., Gaggini M., Castoldi A., Reghellin V., Nonnis S. et al. (2015). Snf1 phosphorylates adenylate cyclase and negatively regulates protein kinase A-dependent transcription in *Saccharomyces cerevisiae*. *J. Biol. Chem.* 290, 24715-24726. 10.1074/jbc.M115.658005 26309257 PMC4598984

[JCS232124C38] Nickel W., Malsam J., Gorgas K., Ravazzola M., Jenne N., Helms J. B. and Wieland F. T. (1998). Uptake by COPI-coated vesicles of both anterograde and retrograde cargo is inhibited by GTPgammaS in vitro. *J. Cell Sci.* 111, 3081-3090. 9739081 10.1242/jcs.111.20.3081

[JCS232124C39] Nie Z. and Randazzo P. A. (2006). Arf GAPs and membrane traffic. *J. Cell Sci.* 119, 1203-1211. 10.1242/jcs.02924 16554436

[JCS232124C40] Pellett P. A., Dietrich F., Bewersdorf J., Rothman J. E. and Lavieu G. (2013). Inter-Golgi transport mediated by COPI-containing vesicles carrying small cargoes. *Elife* 2, e01296 10.7554/eLife.01296 24137546 PMC3787390

[JCS232124C41] Pevzner I., Strating J., Lifshitz L., Parnis A., Glaser F., Herrmann A., Brügger B., Wieland F. and Cassel D. (2012). Distinct role of subcomplexes of the COPI coat in the regulation of ArfGAP2 activity. *Traffic* 13, 849-856. 10.1111/j.1600-0854.2012.01349.x 22375848

[JCS232124C42] Poon P. P., Cassel D., Spang A., Rotman M., Pick E., Singer R. A. and Johnston G. C. (1999). Retrograde transport from the yeast Golgi is mediated by two ARF GAP proteins with overlapping function. *EMBO J.* 18, 555-564. 10.1093/emboj/18.3.555 9927415 PMC1171148

[JCS232124C43] Poon P. P., Nothwehr S. F., Singer R. A. and Johnston G. C. (2001). The Gcs1 and Age2 ArfGAP proteins provide overlapping essential function for transport from the yeast trans-Golgi network. *J. Cell Biol.* 155, 1239-1250. 10.1083/jcb.200108075 11756474 PMC2199332

[JCS232124C44] Presley J. F., Ward T. H., Pfeifer A. C., Siggia E. D., Phair R. D. and Lippincott-Schwartz J. (2002). Dissection of COPI and Arf1 dynamics in vivo and role in Golgi membrane transport. *Nature* 417, 187-193. 10.1038/417187a 12000962

[JCS232124C45] Puertollano R., Randazzo P. A., Presley J. F., Hartnell L. M. and Bonifacino J. S. (2001). The GGAs promote ARF-dependent recruitment of clathrin to the TGN. *Cell* 105, 93-102. 10.1016/S0092-8674(01)00299-9 11301005

[JCS232124C46] Randazzo P. A. and Hirsch D. S. (2004). Arf GAPs: multifunctional proteins that regulate membrane traffic and actin remodelling. *Cell. Signal.* 16, 401-413. 10.1016/j.cellsig.2003.09.012 14709330

[JCS232124C47] Randazzo P. A. and Kahn R. A. (1994). GTP hydrolysis by ADP-ribosylation factor is dependent on both an ADP-ribosylation factor GTPase-activating protein and acid phospholipids. *J. Biol. Chem.* 269, 10758-10763. 8144664

[JCS232124C48] Rawet M., Levi-Tal S., Szafer-Glusman E., Parnis A. and Cassel D. (2010). ArfGAP1 interacts with coat proteins through tryptophan-based motifs. *Biochem. Biophys. Res. Commun.* 394, 553-557. 10.1016/j.bbrc.2010.03.017 20211604

[JCS232124C49] Rein U., Andag U., Duden R., Schmitt H. D. and Spang A. (2002). ARF-GAP-mediated interaction between the ER-Golgi v-SNAREs and the COPI coat. *J. Cell Biol.* 157, 395-404. 10.1083/jcb.200112092 11970962 PMC2173288

[JCS232124C50] Rhiel M., Hessling B., Gao Q., Hellwig A., Adolf F. and Wieland F. T. (2018). Core proteome and architecture of COPI vesicles. *bioRxiv* 254052 10.1101/254052

[JCS232124C51] Robinson M., Poon P. P., Schindler C., Murray L. E., Kama R., Gabriely G., Singer R. A., Spang A., Johnston G. C. and Gerst J. E. (2006). The Gcs1 Arf-GAP mediates Snc1,2 v-SNARE retrieval to the Golgi in yeast. *Mol. Biol. Cell* 17, 1845-1858. 10.1091/mbc.e05-09-0832 16452633 PMC1415299

[JCS232124C74] Ruiz A., Liu Y., Xu X. and Carlson M. (2012). Heterotrimer-independent regulation of activation-loop phosphorylation of Snf1 protein kinase involves two protein phosphatases. National Academy of Sciences. *Proc. Natl. Acad. Sci. USA.* 109, 8652-8657. 10.1073/pnas.1206280109 22589305 PMC3365208

[JCS232124C52] Schindler C. and Spang A. (2007). Interaction of SNAREs with ArfGAPs Precedes Recruitment of Sec18p/NSF. *Mol. Biol. Cell* 18, 2852-2863. 10.1091/mbc.e06-08-0756 17522384 PMC1949378

[JCS232124C53] Schindler C., Rodriguez F., Poon P. P., Singer R. A., Johnston G. C. and Spang A. (2009). The GAP domain and the SNARE, coatomer and cargo interaction region of the ArfGAP2/3 Glo3 are sufficient for Glo3 function. *Traffic* 10, 1362-1375. 10.1111/j.1600-0854.2009.00952.x 19602196

[JCS232124C54] Shibuya A., Margulis N., Christiano R., Walther T. C. and Barlowe C. (2015). The Erv41-Erv46 complex serves as a retrograde receptor to retrieve escaped ER proteins. *J. Cell Biol.* 208, 197-209. 10.1083/jcb.201408024 25583996 PMC4298680

[JCS232124C55] Spang A. and Schekman R. (1998). Reconstitution of retrograde transport from the Golgi to the ER in vitro. *J. Cell Biol.* 143, 589-599. 10.1083/jcb.143.3.589 9813082 PMC2148153

[JCS232124C56] Spang A., Shiba Y. and Randazzo P. A. (2010). Arf GAPs: gatekeepers of vesicle generation. *FEBS Lett.* 584, 2646-2651. 10.1016/j.febslet.2010.04.005 20394747 PMC2878913

[JCS232124C57] Springer S., Spang A. and Schekman R. (1999). A primer on vesicle budding. *Cell* 97, 145-148. 10.1016/S0092-8674(00)80722-9 10219233

[JCS232124C58] Suckling R. J., Poon P. P., Travis S. M., Majoul I. V., Hughson F. M., Evans P. R., Duden R. and Owen D. J. (2014). The structural basis for the binding of tryptophan-based motifs by δ-COP. *Proc. Natl. Acad. Sci. USA* 112, 14242-14247. 10.1073/pnas.1506186112 PMC4655537 26578768

[JCS232124C59] Szafer E., Rotman M. and Cassel D. (2001). Regulation of GTP hydrolysis on ADP-ribosylation factor-1 at the Golgi membrane. *J. Biol. Chem.* 276, 47834-47839. 10.1074/jbc.M106000200 11592960

[JCS232124C60] Tanigawa G., Orci L., Amherdt M., Ravazzola M., Helms J. B. and Rothman J. E. (1993). Hydrolysis of bound GTP by ARF protein triggers uncoating of Golgi-derived COP-coated vesicles. *J. Cell Biol.* 123, 1365-1371. 10.1083/jcb.123.6.1365 8253837 PMC2290881

[JCS232124C61] Watson P. J., Frigerio G., Collins B. M., Duden R. and Owen D. J. (2004). gamma-COP Appendage Domain - Structure and Function. *Traffic* 5, 79-88. 10.1111/j.1600-0854.2004.00158.x 14690497

[JCS232124C63] Weimer C., Beck R., Eckert P., Reckmann I., Moelleken J., Brügger B. and Wieland F. (2008). Differential roles of ArfGAP1, ArfGAP2, and ArfGAP3 in COPI trafficking. *J. Cell Biol.* 183, 725-735. 10.1083/jcb.200806140 19015319 PMC2582887

[JCS232124C64] Xu P., Baldridge R. D., Chi R. J., Burd C. G. and Graham T. R. (2013). Phosphatidylserine flipping enhances membrane curvature and negative charge required for vesicular transport. *J. Cell Biol.* 202, 875-886. 10.1083/jcb.201305094 24019533 PMC3776346

[JCS232124C65] Xu P., Hankins H. M., MacDonald C., Erlinger S. J., Frazier M. N., Diab N. S., Piper R. C., Jackson L. P., MacGurn J. A. and Graham T. R. (2017). COPI mediates recycling of an exocytic SNARE by recognition of a ubiquitin sorting signal. *Elife* 6, e28342 10.7554/eLife.28342 29058666 PMC5663479

[JCS232124C66] Yahara N., Sato K. and Nakano A. (2006). The Arf1p GTPase-activating protein Glo3p executes its regulatory function through a conserved repeat motif at its C-terminus. *J. Cell Sci.* 119, 2604-2612. 10.1242/jcs.02997 16735437

[JCS232124C67] Yanagisawa L. L., Marchena J., Xie Z., Li X., Poon P. P., Singer R. A., Johnston G. C., Randazzo P. A. and Bankaitis V. A. (2002). Activity of specific lipid-regulated ADP ribosylation factor-GTPase-activating proteins is required for Sec14p-dependent Golgi secretory function in yeast. *Mol. Biol. Cell* 13, 2193-2206. 10.1091/mbc.01-11-0563 12134061 PMC117305

[JCS232124C68] Yang J.-S., Lee S. Y., Gao M., Bourgoin S., Randazzo P. A., Premont R. T. and Hsu V. W. (2002). ARFGAP1 promotes the formation of COPI vesicles, suggesting function as a component of the coat. *J. Cell Biol.* 159, 69-78. 10.1083/jcb.200206015 12379802 PMC2173491

[JCS232124C69] Yang J.-S., Hsu J.-W., Park S.-Y., Li J., Oldham W. M., Beznoussenko G. V., Mironov A. A., Loscalzo J. and Hsu V. W. (2018). GAPDH inhibits intracellular pathways during starvation for cellular energy homeostasis. *Nature* 561, 263-267. 10.1038/s41586-018-0475-6 30209366 PMC6152935

[JCS232124C70] Yoshihisa T., Barlowe C. and Schekman R. (1993). Requirement for a GTPase-activating protein in vesicle budding from the endoplasmic reticulum. *Science* 259, 1466-1468. 10.1126/science.8451644 8451644

[JCS232124C71] Yu X., Breitman M. and Goldberg J. (2012). A structure-based mechanism for Arf1-dependent recruitment of coatomer to membranes. *Cell* 148, 530-542. 10.1016/j.cell.2012.01.015 22304919 PMC3285272

