# Supplementary material for: Dissection of GTPase-activating proteins reveals functional asymmetry in the COPI coat of budding yeast
Source: J Cell Sci. 2019 Aug 29;132(16):jcs232124. doi: 10.1242/jcs.232124 (PMC6737914; doi:10.1242/jcs.232124)
Supplement: Supplementary information [file joces-132-232124-s1.pdf]

Table S1. Yeast strains used in this study

No.	Strain	Genotype	Source
1.	BY4741	*MAT**a** his3Δ0 leu2Δ0 met15Δ0 ura3Δ0*	Arakel et al. 2016
2.	Ret2LD2α	*(BY4743 Spore) MAT**a** his3Δ1 leu2Δ0 met15Δ0 ura3Δ0; YFR051c::kanMX4; p415 ret2LD2α*	Arakel et al. 2016
3.	Ret2LD	*(BY4743 Spore) MAT**a** his3Δ1 leu2Δ0 met15Δ0 ura3Δ0; YFR051c::kanMX4; p415 ret2LD*	Arakel et al. 2016
4.	Δ*gcs1*	*MAT**a** his3Δ0 leu2Δ0 met15Δ0 ura3Δ0; YDL226C::CloNAT*	This study
5.	Δ*glo3*	*MAT**a** his3Δ0 leu2Δ0 met15Δ0 ura3Δ0; YER122C::CloNAT*	This study
		*MAT**a** his3-11,15 leu2, trp1, ura3, ade2 glo3::HIS3*	Estrada et al., 2014
6.	*GFP-Glo3*	*MAT**a** his3Δ0 leu2Δ0 met15Δ0 ura3Δ0; Nop1prom-GFP- YER122C:: ura3*	This study
7.	Δ*gcs1* *GFP Glo3*	*MAT**a** his3Δ0 leu2Δ0 met15Δ0 ura3Δ0; YDL226C::CloNAT; Nop1prom-GFP- YER122C:: ura3*	This study
8.	*sec26FW* *(F856A, W860A)*	*MAT**a** his3-11,15 leu2, trp1, ura3, ade2*	This study
9.	YAS4326 Snf1K84R-HA	*MATa Snf1::Snf1K84R-3HA(TRP) leu2,3, his 3-11, trp1-Δ1 ura3-1 ade2-1*	This study
10.	YAS3787 Δ*snf1*	*MATa Snf1::His3 leu2,3, his 3-11, trp1-Δ1 ura3-1 ade2-1*	This study
11.	YAS3098	*MATa Snf1:: Snf1-3HA(TRP) leu2,3, his 3-11, trp1-Δ1 ura3-1 ade2-1*	This study
12.	YAS4516	*MATa ARF1::ARF1-yEGFP (kanMX), VRG4::VRG4-mCherry (hphNTI) glo3::URA3 leu2-Δ1, lys2-801 ade2-101c his3-Δ200 p415 Glo3*	This study

Table S2. Plasmids used in this study

All plasmids used in this study from the Schwappach lab have been deposited with the Addgene plasmid repository. A detailed description of these plasmids are available at: https://www.addgene.org/Blanche_Schwappach/

No.	Plasmids	Addgene ID	Database ID
1.	p415 Gcs1	112646	AU2328
2.	p415 Gcs1 R54K	112647	AU2329
3.	p415 Gcs1 R54K L246D	112648	AU2330
4.	p415 Gcs1 R54K AxxA	112649	AU2331
5.	p415 Gcs1 Δ3xF AxxA	112650	AU2332
6.	p415 Glo3	112651	AU2333
7.	p415 Glo3 R59K	112652	AU2334
8.	p415 Glo3 R59K ΔC	112653	AU2335
9.	p415 Glo3 R59K ΔGRM-ΔC	112654	AU2336
10.	p415 Glo3 R59K Δ2x+ve	112655	AU2337
11.	p415 Glo3 R59K S389,398 A	112656	AU2338
12.	p415 Glo3 R59K S389,398 D	112657	AU2339
13.	p415 GFP-Glo3	112658	AU2340
14.	p415 GFP-Glo3 ΔGRM-ΔC	112659	AU2341
15.	p415 GFP-Glo3 S389,398 A	112660	AU2342
16.	p415 GFP-Glo3 S389,398 D	112661	AU2343
17.	p415 GFP-Glo3 Δ2x+ve	112662	AU2344
18.	p415 Met25 Glo3 ΔGRM-ΔC	129487	
19.	p415 Met25 Glo3 S389,398 A	129485	
20.	p415 Met25 Glo3 S389,398 D	129486	
21.	A102 GAP-BoCCS-GRM	123286	
22.	A102 GAP-BoCCS	123287	
23.	A102 BoCCS-GRM	123288	
24.	A102 par-BoCCS-GRM	123289	
25.	A102 BoCCS	123290	
26.	A102 GRM	123291	
27.	A102 Glo3 Δ AmpH Δ2x+ve	129484	
28.	p425TEF Glo3-FLAG		
29.	p425TEF Glo3-FLAG S389D		
30.	p425TEF Glo3-FLAG SS389/390AA		

Journal of Cell Science • Supplementary information

Table S3. Proteins co-purifying with Glo3 and Gcs1.

List of complete protein identification results from LC-MS/MS analysis of affinity purified Glo3 and Gcs1. Contains spectral counts of identified proteins and their UniProt accession identities. See Excel file.

Click here to Download Table S3

Table S4. Arf1-GFP FRAP parameters.

Auxiliary to Fig. 4D and 4E.

Kinetic parameters derived from the FRAP data obtained by FRAP analysis of Arf1-GFP turnover at the Golgi. Mean with 95% confidence interval for dissociation rate k_{off} and mobile fraction F_m are shown.

**Protein analyzed**	**Strain background**	**D**	**Dissociation rate k_{off} [s^{-1}]**		**Mobile fraction F_m [%]**	
			mean	95% CI	mean	95% CI
**Arf1 GFP**	WT	+	0.164	0.154 to 0.173	0.769	0.761 to 0.776
	WT	-	0.234	0.219 to 0.249	0.854	0.849 to 0.859
	Δglo3 +EV	+	0.198	0.182 to 0.213	0.713	0.707 to 0.719
	Δglo3 +Glo3 WT	+	0.209	0.192 to 0.226	0.706	0.701 to 0.712
	Δglo3 +Glo3 AA	+	0.283	0.258 to 0.308	0.622	0.618 to 0.627
	Δglo3 +Glo3 DD	+	0.196	0.177 to 0.215	0.765	0.756 to 0.773

Calculated parameters derived from FRAP analysis of Arf1 GFP at the cis-Golgi compartment. CI – confidence interval. D – Glucose.

Table S5. Antibodies used in this study

Unique Identifier	Antibody		Species	Source
**Ab0559**	Coat (COPI)	Polyclonal	Rabbit	Hans Dieter Schmitt, MPI for Biophysical Chemistry, Germany
**Ab0172**	GFP	Polyclonal	Rabbit	Torrey Pines biolabs (TP401)
**Ab0234**	MBP	Monoclonal	Mouse	New England BioLabs (E8032S)
**Ab0180**	Glo3	Polyclonal	Rabbit	Anne Spang, Biozentrum University of Basel, Switzerland.
**Ab0162**	Gcs1	Polyclonal	Rabbit	Anne Spang, Biozentrum University of Basel, Switzerland.
**Ab0270**	Pgk1	Monoclonal	Mouse	Thermo Fisher Scientific (459250)

Journal of Cell Science • Supplementary information

Fig. S1. COPI binds the BoCCS domain of Glo3.

Related to Figure 2

Binding of TAP-purified coatomer to MBP fusion proteins of Glo3. The bound fraction was eluted and analysed by SDS/PAGE. Western blots were detected with a coat antiserum recognising five of the seven coatomer subunits. Glo3 Δ2x+ve – alanine substitution of COPI binding region within the BoCCS domain.

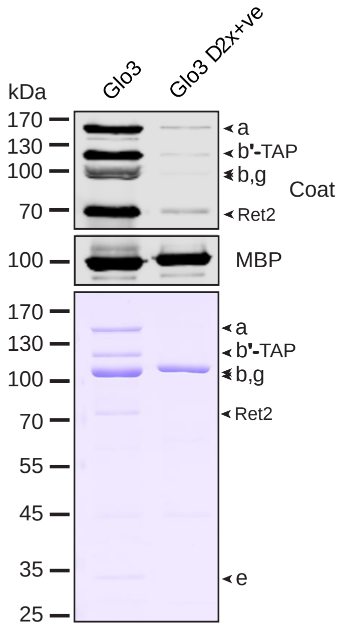

Journal of Cell Science • Supplementary information

Fig. S2. Deletion of the Glo3 C-terminal amphipathic helix does not perturb its intracellular localization or its association with COPI.

Related to Figure 3

(A) Affinity chromatography of GFP-tagged Glo3 variants from detergent extracts of Δ*glo3* strains harbouring the indicated GFP-tagged constructs and subsequent evaluation of COPI association. Western blots were detected with a coat antiserum recognising five of the seven coatomer subunits.

(B) Steady-state localisation analysis of GFP tagged proteins (expressed under the *Met25* promoter) in a Δ*glo3* strain. Scale bar, 5 μm.

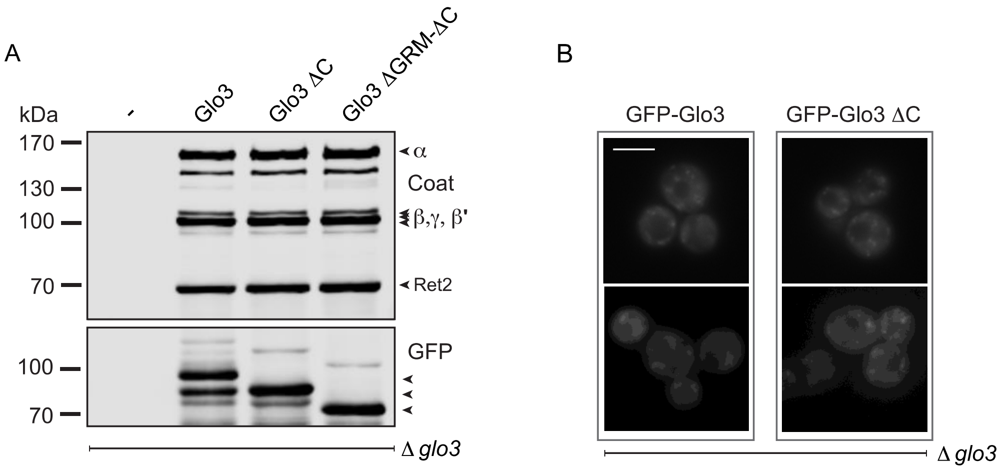

Journal of Cell Science • Supplementary information

Fig. S3. S389 of the GRM domain is phosphorylated by the Snf1 Kinase. Related to Figure 4

(A) Schematic representation of the Glo3 variants used in (B and C).

(B) Immunoprecipitation of HA-tagged Snf1 for use in (C).

(C) Kinase assay demonstrating direct phosphorylation of Glo3 by Snf1. Purified Glo3 and variants thereof were incubated in the presence of Snf1, a kinase dead Snf1 mutant (K84R) or in its absence, subjected to SDS-PAGE and detected by autoradiography.

(D) Quantification of S389 phosphorylation in the presence or absence of the Snf1 kinase using a S398A construct.

(E) Quantification of S398 phosphorylation in the presence or absence of the Snf1 kinase using a S389A construct.

(F) Growth assay. Growth of *sec26[FW]* *Δglo3* strains harbouring the indicated constructs.

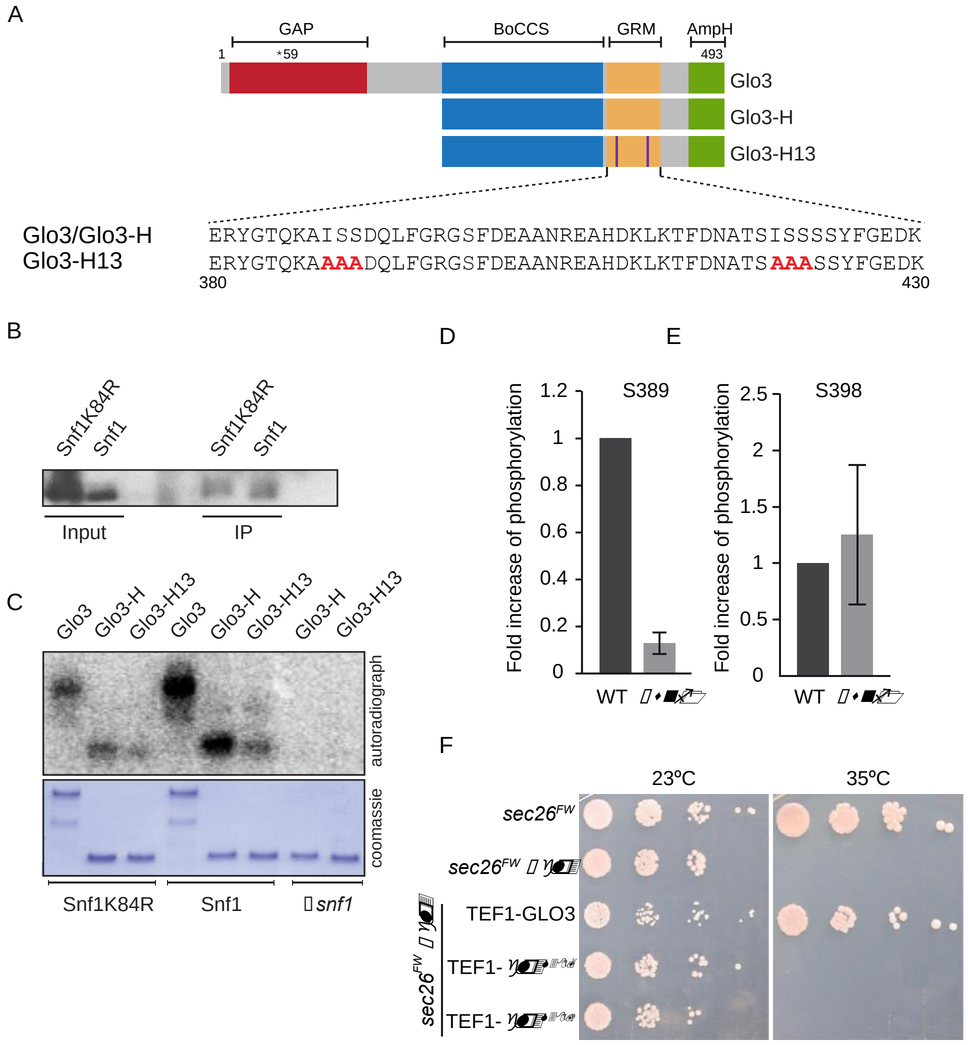

Journal of Cell Science • Supplementary information

Fig S4. Phosphorylation/ dephosphorylation of the Glo3 GRM domain alters Arf1 dynamics on Golgi membranes.

Related to Figure 4

Live cell imaging of C-terminally GFP tagged Arf1 and C-terminally mCherry tagged Vrg4 in a Δ*glo3* strain expressing the indicated constructs. Arrows indicate fields/regions of co-localisation of Arf1 and the Golgi marker Vrg4 used for fluorescence recovery after photobleaching (FRAP) experiments to study the dynamics of Arf1 at the Golgi. Scale bar, 5 µm.

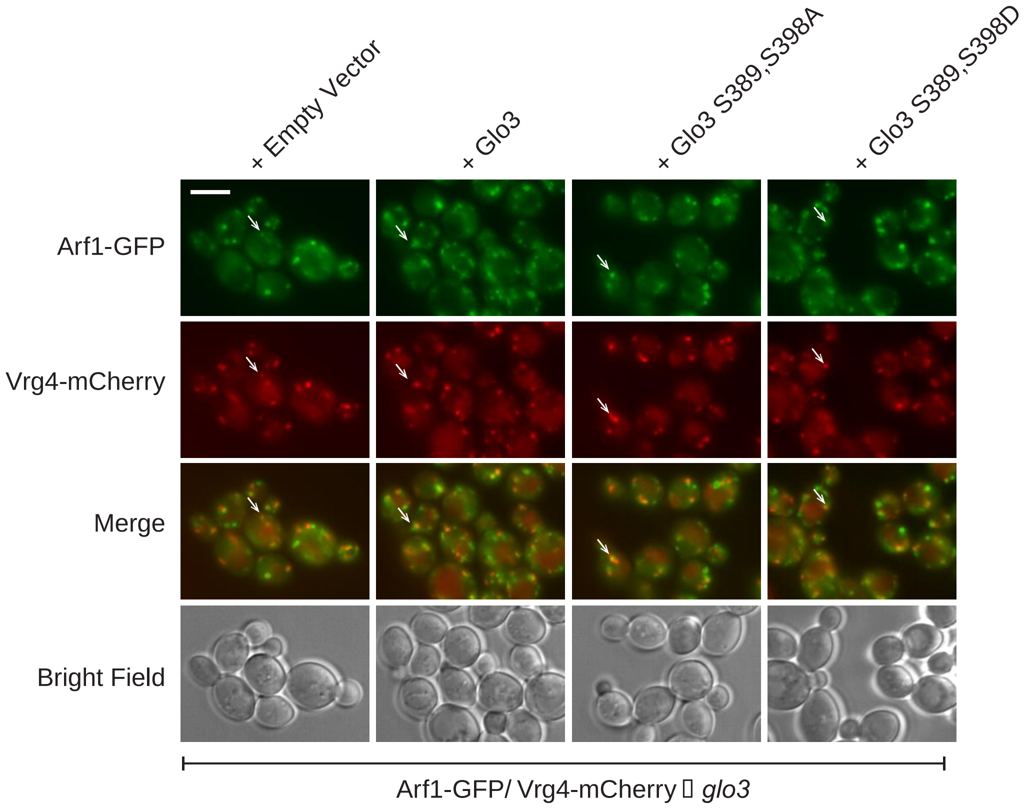

Journal of Cell Science • Supplementary information
